# Identification and functional characterisation of a locus for target site integration in *Fusarium* *graminearum*

**DOI:** 10.1186/s40694-024-00171-8

**Published:** 2024-02-26

**Authors:** Martin Darino, Martin Urban, Navneet Kaur, Ana Machado Wood, Mike Grimwade-Mann, Dan Smith, Andrew Beacham, Kim Hammond-Kosack

**Affiliations:** 1 Protecting Crops and the Environment, Rothamsted Research, Harpenden, Hertfordshire AL5 2JQ UK; 2 Intelligent Data Ecosystems, Harpenden, Hertfordshire AL5 2JQ UK; 3 Jealott’s Hill International Research Centre, Syngenta, Warfield, Bracknell, RG42 6EY UK; 4 Human Milk Foundation, Daniel Hall Building, Harpenden, Hertfordshire AL5 2JQ UK; 5 Centre for Crop and Environment Sciences, Harper Adams University, Shropshire, TF10 8NB UK

**Keywords:** *Fusariumgraminearum*, Fusarium Head Blight, Fungal transformation, Target site integration, Complementation, Secretion, Coleoptiles, Wheat, Confocal microscopy, Genome sequence

## Abstract

**Background:**

Fusarium Head Blight (FHB) is a destructive floral disease of different cereal crops. The Ascomycete fungus *Fusarium* *graminearum* (*Fg*) is one of the main causal agents of FHB in wheat and barley. The role(s) in virulence of *Fg* genes include genetic studies that involve the transformation of the fungus with different expression cassettes. We have observed in several studies where *Fg* genes functions were characterised that integration of expression cassettes occurred randomly. Random insertion of a cassette may disrupt gene expression and/or protein functions and hence the overall conclusion of the study. Target site integration (TSI) is an approach that consists of identifying a chromosomal region where the cassette can be inserted. The identification of a suitable locus for TSI in *Fg* would avert the potential risks of ectopic integration.

**Results:**

Here, we identified a highly conserved intergenic region on chromosome 1 suitable for TSI. We named this intergenic region TSI locus 1**.** We developed an efficient cloning vector system based on the Golden Gate method to clone different expression cassettes for use in combination with TSI locus 1. We present evidence that integrations in the TSI locus 1 affects neither fungal virulence nor fungal growth under different stress conditions. Integrations at the TSI locus 1 resulted in the expression of different gene fusions. In addition, the activities of *Fg* native promoters were not altered by integration into the TSI locus 1. We have developed a bespoke bioinformatic pipeline to analyse the existence of ectopic integrations, cassette truncations and tandem insertions of the cassette that may occurred during the transformation process. Finally, we established a protocol to study protein secretion in wheat coleoptiles using confocal microscopy and the TSI locus 1.

**Conclusion:**

The TSI locus 1 can be used in *Fg* and potentially other cereal infecting Fusarium species for diverse studies including promoter activity analysis, protein secretion, protein localisation studies and gene complementation. The bespoke bioinformatic pipeline developed in this work together with PCR amplification of the insert could be an alternative to Southern blotting, the gold standard technique used to identify ectopic integrations, cassette truncations and tandem insertions in fungal transformation.

**Supplementary Information:**

The online version contains supplementary material available at 10.1186/s40694-024-00171-8.

## Background

Fusarium Head Blight (FHB) is a destructive floral disease of different cereal crops such as wheat, barley, maize and oat [1, 2]. The Ascomycete fungus *Fusarium* *graminearum* (*Fg*) is one of the main causal agents of FHB in wheat and barley crops in Europe, Asia and America [3]. The disease is characterised by reducing grain quality and safety. During infection, *Fg* produces a diverse repertoire of mycotoxins where deoxynivalenol is one of the most frequent detected in cereal grains [4]. Contamination of grains with different mycotoxins make the crop unsuitable for human and/or animal consumption [4]. Due to the ever growing worldwide economic and societal relevance of FHB disease, the role(s) in virulence of 1571 *Fg* genes, i.e. 11% of the predicted *Fg* gene repertoire, has been formally tested, described in different peer reviewed studies and then manually curated into the multispecies PHI-base database [5]. These studies often include approaches such as gene deletion, gene complementation, promoter expression and protein localisation to characterise a gene and/or a protein function [6–8]. Gene complementation and protein localisation involve the stable transformation of *Fg* with an expression cassette. Integration of the cassette into the genome can occur by either homologous or non-homologous recombination [6, 9]. Non-homologous recombination or ectopic integration happens when the cassette is inserted randomly into the genome. Random insertion of a cassette may disrupt gene expression and/or protein functions and hence the overall conclusion of the study. Target site integration (TSI) is not a new concept in fungal molecular genetic studies. The approach has been applied in fungal plant pathogens where integration of a cassette is possible by homologous recombination such as *Ustilago* *maydis* (*U.* *maydis*) and *Magnaporthe* *oryzae* (*M.* *oryzae*) [10, 11]. The approach consists of identifying a chromosomal region where the cassette can be inserted by homologous recombination. A suitable locus for TSI is defined as a region where insertion of an expression cassette does not alter the growth and virulence of the pathogen. In addition, the region should be transcriptionally active to allow proper expression of the cassette. We have observed in several studies where *Fg* genes functions were characterised that integration of expression cassettes occurred randomly [7, 9, 12]. Integrations for *Fg* gene complementation and protein localisation studies are usually done by ectopic integration. The identification of a suitable locus for TSI in *Fg* would avert the potential risks of ectopic integration. To identify new virulence factors in *Fg*, Beacham and collaborators developed a bespoke bioinformatic approach that allowed the identification of a micro-region in chromosome 1 enriched in homologues of known virulence genes from multiple cereal and non-cereal disease-causing fungal species [13]. These virulence genes had been manually curated over a 10-year period into the publicly available pathogen-host interactions database (PHI-base) [13–15]. The micro-region spanned the region from FGRAMPH1_01G06783 to FGRAMPH1_01G06811 and contains a total of 15 genes. This micro-region contained homologues of five already well characterised fungal virulence genes. In addition, the micro-region was described as being transcriptionally active, residing in a region of low recombination and highly conserved among different Fusarium species such as *Fusarium* *verticillioides*, *Fusarium* *oxysporum* and *Fusarium* *solani* [13, 16]. These three characteristics make this genomic interval potentially suitable for target site integration. Immediately adjacent to this micro region, we have identified a 2.7 kb intergenic region suitable for TSI where an expression cassette can be inserted. We named this intergenic region the TSI locus 1. We developed an efficient cloning vector system based on the Golden Gate method to clone different expression cassettes for use in combination with TSI locus 1. We present evidence that integrations in the TSI locus 1 affects neither fungal virulence, fungal growth under different stress conditions nor expression of the genes flanking the TSI locus 1. Integrations at the TSI locus 1 successfully resulted in the expression of different gene fusions. In addition, the activities of the trichodiene synthase promoter and an effector promoter were not altered by integration into the TSI locus 1. We have developed a bespoke bioinformatic pipeline to analyse the existence of deletions, ectopic integrations, cassette truncations and tandem insertions of the cassette that may occur during the transformation process. Finally, we established a protocol to study protein secretion in wheat coleoptiles using confocal microscopy and used the TSI locus 1 for stable expression of different gene fusions. In summary, the TSI locus 1 can be used in *Fg* for diverse studies including promoter activity analysis,  protein secretion, protein localisation studies and gene complementation.

## Methods

### Strains, media and culture

*Fg* wild strain PH-1 [16] was used for all the transformation events whilst for the complementation analysis the PH-1*-*Δ*osp24-1* mutant was used. Fungal strains were maintained on SNA (synthetic nutrient poor agar) plates. For growth and sporulation of the strains transformed into the TSI locus 1 or *osp24* locus, the SNA plates also contained either 75 µg/mL of geneticin (G148, Sigma-Aldrich, Germany) or 75 µg/mL of hygromycin B (Calbiochem, Germany), respectively as the selection agent. Plates were kept under constant illumination (UV and white light) at room temperature (RT). Conidia production on SNA plate was induced by adding 4 mL of TB3 media (3 g/L yeast extract, 3 g/L Casamino acids, 200 g sucrose/L) to 7 days-old mycelia [17]. Spores were collected after 24 h in sterile water and stored at – 80 ℃ as described before [17]. DNA plasmids were amplified using *Escherichia* *coli* strain DH5ɑ. *E.* *coli* transformed cells were selected in Luria–Bertani (LB) agar media containing ampicillin 100 µg/mL (Melford, UK) or spectinomycin 150 µg/mL (Melford, UK).

Defects in radial growth in the transformant strains compared to PH-1 were evaluated under different stress conditions. Twenty-five mL of half-strength PDA (Potato Dextrose Agar) containing 2% agar were mixed with different stress inducing agents such as salt stress (1 M NaCl), and membrane stresses (100 µg/mL Calcofluor, 50 µg/mL Congo Red, 0.02% Tergitol or 0.002% SDS). The agar mixed with a single stress inducer was poured into squares plates (Grenier Bio-One, UK). Serial spore dilutions were prepared from water stocks containing 10^6^ conidia/mL. From each transformant and PH-1, 5 µL of each spore dilution was plated. A half-strength PDA plate without any stress agent was included as the control. Plates were incubated in a dark cabinet at RT for the entire experiment. Photographs were taken 3 days post inoculation (dpi). The experiment was repeated three times.

### Plasmids design and cloning strategies

To build the Fg vector, several cloning steps were performed (Additional file 1: Fig. S1A). First, to generate a geneticin resistance cassette, the *gpdA* promoter (P_gpdA_) and the *trpC* terminator (T_trpC_) were synthesised (Epoch Life Science, US). The *geneticin* gene was amplified from the pCGEN vector [18]. Next, BsaI sites to the P_gpdA_, T_trpC_ and *geneticin* gene were added using primer combinations pGPDApro_F–pGPDApro_R, TtrpC_F–TtrpC_R, and Gene_F1–Gene_R1, respectively. To assemble the geneticin cassette (P_gpdA_-*geneticin*-T_trpC_), the Golden Gate protocol was used as described before [19]. Finally, from the geneticin cassette, a PCR product was amplified containing the P_gpdA_ and a split fragment of the *geneticin* gene (P_gpdA_*-geneticin*_1-664_) with primers Gene_R XhoI and Gene_F BsaI. The right border (RB) of the TSI locus 1 was cloned from PH-1 genomic DNA using primers FgRB_R SapI (P4) and FgRB_F XhoI. The PCR products containing the RB border and the P_gpdA_*-geneticin*_1-664_ were digested with XhoI (New England Biolabs, UK) and ligated using T4 DNA ligase (New England Biolabs, UK). The ligation product (*geneticin*_1-664_-P_gpdA_*-*RB) was amplified by PCR with primers Gene_F SapI (P3) and FgRB_R SapI (P4).

The spectinomycin cassette and the bacterial origin of replication (SpecR-Ori) were amplified by PCR from the pGreen vector [20] with primers Dest_F SapI and Dest_R SapI. To assemble the entire Fg vector, the PCR products containing the *geneticin*_1-664_-P_gpdA_*-*RB and the SpecR-Ori were digested with SapI (New England Biolabs, UK) and ligated with T4 DNA ligase. *E.* *coli* was transformed with the product of the restriction-ligation reaction. The correct clone was selected by sequencing the vector.

To build the vector pJET-LB-geneticin, the following steps were performed (Additional file 1: Fig. S1B). From the geneticin cassette a PCR product containing a split fragment of the *geneticin* gene and the terminator (*geneticin*_128-795_-T_trpC_) was amplified with primers TtrpC_F AgeI and Gene_R2 BsaI. The LB border of the TSI locus 1 was amplified by PCR from PH-1 genomic DNA with primers FgLB_F1 and FgLB AgeI_R. The PCR products containing the *geneticin*_128-795_-T_trpC_ and LB border were digested using AgeI (New England Biolabs, UK) and ligated. The ligation product (LB-T_trpC_-*geneticin*_795-128_) was amplified by PCR using primers FgLB_F XhoI (P1) and Gene_R XhoI (P2). Finally, the PCR product was cloned into the vector pJET (Thermo Fisher Scientific, UK) following the manufacturer’s instructions. Positive clones were selected by sequencing.

Promoter regions were cloned from PH-1 genomic DNA. We cloned 1000 bp upstream of the start codon of the trichodiene synthase (*Tri5*) and FGRAMPH1_01G11655 (*Fgeffector1*) genes with primer combinations ProTri5_F1–ProTri5_R2 and ProFgEffector1_F–ProFgEffector1_R, respectively. The *Tri5* promoter (P_Tri5_) possesses an internal BsaI site. To mutate the site (T_-219_A), two fragments were amplified from the cloned promoter region using primer combinations ProTri5_F1–ProTri5_R1 and ProTri5_F2–ProTri5_R2. Next, the Golden Gate protocol was used to mutate the site. The product of the Golden Gate reaction was amplified with primers ProTri5_F1 and ProTri5_R2 and used for cloning into the Fg vector. The *trpC* promoter (P_trpC_) was cloned from plasmid pHYG1.4 [21] with primers PtrpC_F and PtrpC_R. Promoters P_trpC_ and P_gpdA_, and terminator T_trpC_ containing BsaI sites were cloned into vector pJET as described.

To clone the coding sequence of *Fgeffector1*, cDNA from wheat floral tissue infected with PH-1 was used as the template with primers FgEffector1_F and FgEffector1_R.

Constructs cloned into the Fg vector were done using the Golden Gate protocol and the library of modules as described [19]. All PCR amplifications were done using Q5® High-Fidelity DNA Polymerase (New England Biolabs, UK) following manufacturer’s instructions. All primers used in this study are listed in Additional file 2: Table S1.

### Deletion of *osp24* gene and complementation at TSI locus 1

To study complementation at TSI locus 1, the identified secreted virulence protein coded by the orphan secreted protein 24 (*osp24*) gene [22] was deleted in PH-1 using the ‘split-marker’ approach [23]. To delete the *osp24* gene, two vectors (pMU487 and pMU488) were designed. Vector pMU487 consists of a DNA fragment containing 1000 bp upstream the start codon of *osp24* gene (P_osp24_) followed by the partial sequence of the hygromycin B (Hyg) cassette (P_trpC_-Hyg_1-761_). Vector pMU488 consists of a fragment of the Hyg cassette (Hyg_296-1027_) followed by 504 bp of the terminator region of *osp24* gene (T_osp24_). Both vectors shared a 466 bp overlapping region of the Hyg cassette to facilitate recombination. To construct these vectors, P_trpC_-Hyg_1-761_ and Hyg_296-1027_ fragments were PCR amplified from pHyg1.4 vector [21] using primer combinations O3–O4 and O5–O6, respectively. P_osp24_ and T_osp24_ sequences were amplified from PH-1 genomic DNA using primer combination O1–O2 and O7–O8, respectively. Finally, Gibson assembly was used to fuse the PCR products (P_osp24_ with P_trpC_-Hyg_1-761_ and Hyg_296-1027_ with T_osp24_). The PCR products were ligated into the pGEM-T Easy vector (Promega, UK). Selection of the deleted strain (PH-1-Δ*osp24*-1) was done using the following primer combinations O9–O10, O11–O12 and O13–O14.

The PH-1-Δ*osp24*-1 mutant was complemented at the TSI locus 1 with the *osp24* gene. A DNA fragment containing the P_osp24_*,* the *osp24* coding sequence and the T_osp24_ (P_osp24_*-osp24-*T_osp24_*)* was amplified from genomic PH-1. Two primer combinations: O15–O16 and O17–O18 were used to amplify the fragment due to the presence of an internal BsaI site in the promoter region (T_-313_A). The site was mutated using Golden Gate approach as described above. Finally, the PCR product containing the P_osp24_*-osp24-*T_osp24_ was cloned into the Fg vector.

The vector system as well as Golden Gate modules developed in this work (Table 1) are available from Addgene (https://www.addgene.org/).

**Table 1:** List of vectors developed in this work

Name	Purpose	Bacterial resistance	Addgene ID
Fg vector	Destination vector for cloning and transformation into TSI locus 1	Spectinomycin	213464
pJET-LB-geneticin	Vector to amplify the LB-*geneticin* fragment for transformation into TSI locus 1	Ampicillin	213468
Fg-P_trpC_*-mCherry-*T_trpC_	Constitutive expression of a non-secreted version of mCherry	Spectinomycin	
Fg-P_trpC_*-*SP_osp24_*-mCherry-*T_trpC_	Constitutive expression of a secreted version of mCherry	Spectinomycin	
Fg-P_Tri5_*-GFP-*T_trpC_	GFP expression controlled by the *Tri5* promoter	Spectinomycin	
Fg-P_FgEffector1_*-FgEffector1-GFP-*T_trpC_	Fgeffector1-GFP expression controlled by the *Fgeffector1* promoter	Spectinomycin	
Fg-P_trpC_*-GFP-*T_trpC_	Constitutive expression of a non-secreted version of GFP	Spectinomycin	
Fg-P_osp24_*-osp24-*T_osp24_	*osp24* expression under the *osp24* promoter	Spectinomycin	
pMU487	Vector to amplify the P_osp24_*-*P_trpC_*-HyG*_*1-761*_ fragment for *osp24* gene mutation	Ampicillin	
pMU488	Vector to amplify the *Hyg*_*296-1027*_*-*T_osp24_ fragment for *osp24* gene mutation	Ampicillin	
pJET-P_trpC_	Vector containing the *trpC* promoter for Golden Gate cloning	Ampicillin	213465
pJET-P_gpdA_	Vector containing the *gpdA* promoter for Golden Gate cloning	Ampicillin	213466
pJET-T_trpC_	Vector containing the *trpC* terminator for Golden Gate cloning	Ampicillin	213467

### Fungal transformation

Integrations into the TSI locus 1 were done following an adaptation of the ‘split-marker’ approach previously described [24]. Constructs cloned in the Fg vector were amplified by PCR using primer combination P3 and P4. The region containing the LB and a fragment of the geneticin cassette was amplified by PCR from the vector pJET-LB-geneticin using primer combination P1 and P2. PCR products were amplified using HotStar TAQ polymerase (Qiagen, Germany) following the manufacturer’s instructions. PCR products were adjusted to a concentration of 2 µg/µL. A 5 µL aliquot of each PCR product amplified from the Fg vector was mixed with 5 µL of the product amplified from the vector pJET-LB-geneticin. The mixture of PCR products was used to transform 1 × 10^8^ protoplasts derived from fungal conidia following a previously described protocol [25]. For the *osp24* gene deletion, LB and RB fragments were amplified using U874–U768 and U769–U868 primers from pMU487 and pMU488, respectively.

Transformants were selected in regeneration media (0.7% agarose, 0.2% Yeast Extract, 0.2% Casein-Hydrolysate (N-Z-Amine A), 0.8 M sucrose) containing 75 µg/mL of geneticin. Two days after transformation, six well-spaced transformants were selected and transferred to a 6-well plate containing SNA agar media with 75 µg/mL of geneticin. Aerial hyphal fragments (minus agar) were collected from each transformant, and DNA was extracted using an alkaline-heat DNA extraction protocol. Briefly, hyphae were resuspended in 100 µL of a 50 mM NaOH solution and heated at 95 ℃ for 15 min. Next, 11 µL of 1 M Tris–HCl (pH 7) was added to the mixture and centrifuged to precipitate hyphae debris. Then, a 1 µL aliquot of the suspension was used to validate the transformants by PCR with four primer combinations. Primer combinations P5–P6, P7–P8 and P9–P10 were used to confirm insertion of the expression cassette into the TSI locus 1. Whereas primer combination P11 and P12 was used to test for homozygosity of the transformants. In the case of the complemented PH-1-Δ*osp24* strain, the following primer combinations P5–P6, P7–P8, P11–P12, O9–P10 and O9–O10 were used to test for the correct insertion of the osp24 cassette into the TSI locus 1.

### DNA sequence alignments and whole-genome sequence analysis of transformed strains

Multiple DNA sequence alignments of the LB and RB from PH-1 and various *Fg* isolates and other Fusarium species collected from different global locations were done using Clustal Omega tool [26].

For whole-genome sequence analysis, spores of candidate transformants were inoculated in 200 mL liquid yeast extract peptone dextrose (YPD) complete medium. Spores were grown with agitation (180 rpm) for 2 days at 28 ℃. One gram of fungal biomass was harvested by filtration. DNA was extracted using the Nucleon PhytoPure DNA extraction kit (Cytiva, UK) following manufacturer instructions. Subsequently, the samples were sent to Novogene (Cambridge, UK) for Illumina sequencing. Sequencing was performed using 150-bp paired-end reads, generating 2 G raw data per sample, with PCR-free library preparation. The wildtype strain PH-1 was also included in the sequencing analysis as a control.

To identify the genomic region where the expression cassette was inserted during transformation the following steps were taken. First, a quality control of the reads was assessed by FastQC [27]. Then, adapters and low-quality reads were removed using Trimmomatic [28] with the following trimming steps: ILLUMINACLIP, SLIDINGWINDOW and MINLEN. Reads belonging to each strain were aligned to the reference genome of PH-1 (YL1 version, NCBI GenBank number: PRJNA782099) [29] using HISAT2 [30]. Finally, the read depth at each base of the genome was computed using the option genome coverage from BEDTools [31]. Reads aligned to the reference genome were visualised using Integrative Genomic Viewer (IGV) [32]. Mapping statistics were calculated for each sequenced strain using Qualimap [33]. To identify deletions in the transformant strains, bases with a read depth ≤ 1 were kept using a filter tool from the Galaxy platform (https://usegalaxy.eu/). The filter tool allows the restriction of datasets using simple conditional statements. Bases without coverage may indicate chromosomic regions where deletions or insertions of the expression cassette had occurred.

To identify the existence of truncations and/or tandem insertion that may occur during insertion of the cassette at the TSI locus 1, two *Fg* transformed strains were evaluated. Namely, the PH-1 genome edited by inserting in the TSI locus 1 the expression cassette either from the non-secreted version of mCherry (mCherry) or the secreted version of mCherry (SP-mCherry). Reads from each strain were aligned against the respective genome sequence. Read depth for each base was calculated as described before. The genomic regions containing the different sections of the expression cassette were filtered and for each section an average read depth value was calculated. Ratio values between the average read depth of each section and the average read depth from the two genes (FGRAMPH1_01G06815 and FGRAMPH1_01G06817) flanking the TSI locus 1 were calculated. The ratio values gave an idea about the existence of truncations/tandem insertions present at the TSI locus 1.

Contigs with evidence of truncation/tandem insertions in the mCherry strains were also identified. Unmapped reads after alignment with PH-1 were extracted and a de novo assemble approach was performed using SPAdes with –isolate option [34]. Contigs were blasted against the cassettes of the mCherry and SP-mCherry strains. Contigs containing truncated sequences from the expression cassettes were selected for further analysis. All the tools used to analyse the sequencing data are available at usegalaxy.eu [35]. The raw sequencing data was deposited in the European Nucleotide Archive (ENA) and are accessible through series accession number: PRJEB64490.

### PCR amplification for long amplicons

To amplify the cassette inserted into the TSI locus1, DNA was extracted using the Nucleon PhytoPure DNA extraction kit as described above. Amplification of PCR amplicons were performed using LongRange PCR Kit (Qiagen, Germany). Briefly, 500 ng of good quality genomic DNA (DNA size should be above 15 kb) was used as the template in combination with primer combination P5 and P10. We followed the manufacturer instructions to set a cycling protocol that lasted ~10 h to amplify long PCR products (> 10 kb).

### Growth of strains to test reporter gene expression

To explore reporter gene expression during in vitro growth, liquid cultures were used. These were started by mixing 1 mL of sterile distilled water containing 10^6^ conidia/mL with 9 mL of TB3 media in 50 mL Falcon tubes. Cultures were grown by agitation (100 rpm) at 20 ℃ for 16 h under dark conditions. Then, fluorescence emission from germinated spores was evaluated by confocal microscopy.

### Wheat floral spikes and coleoptiles infection assays

Plants of the susceptible spring wheat cv. Bobwhite or Apogee were grown in a growth chamber as previously described [36]. Wheat plants at the flowering stage were selected for inoculation. We followed the point inoculation protocol [37]. Briefly, wheat spikes at anthesis were inoculated with a 5 µL water solution containing 5 × 10^5^ conidia/mL. Two spikelets per wheat spike were inoculated and five plants per strain. The control plants were inoculated with water only droplets. Point inoculations were done at the 9th and 10th spikelets counted up from the bottom. All inoculated plants were randomised both during the 48 h high humidity incubation and again after placing on the controlled growth room shelf. The growth room conditions were 23 ℃/18 ℃ (day/night), 60% humidity and 16 h photoperiod (180 μmol m^−2^ s^−1^light intensity). At 3 dpi, spikelets showing symptoms of Fusarium infection were selected for confocal analysis. To test whether insertion in the TSI locus 1 affected the infection process, we inoculated the wheat plants with either the wild type PH-1 or the PH-1 strain expressing the non-secreted version of mCherry integrated at the TSI locus 1. A minimum of eight plants per strain were inoculated and at 12 dpi, visibly diseased spikelets were counted below the point of inoculation. The experiment was repeated twice.

Wheat coleoptile infection assays were performed following an adaptation of a previously described protocol [38]. Wheat seeds of the cv. Bobwhite were placed in a 50 mL Falcon tube containing water and vernalised in the dark for 48 h. Small pieces of cotton wool soaked with sterile water were placed in each well of a 24-well tissue culture plate (VWR International, USA). One wheat seed was placed with the crease facing downwards into each well. The plate was placed inside a humidity chamber for 3 days to allow germination and coleoptile elongation. Then, between 2 to 3 mm of the tip of each coleoptile was removed with scissors. A 10 µL clear plastic pipette tip (Starlab, UK) was cut down to 12 mm above the base and a 12 × 15 mm piece of Whatman 1 filter paper (Camlab, UK) was rolled and inserted inside each tip. Each plastic tip was soaked in a solution containing either 5 × 10^5^ conidia/mL sterile distilled water or just sterile distilled water. An individual pipette tip was placed over the top of each cut coleoptile. This step was found to be necessary to keep high humidity conditions during incubation to ensure conidia germination and successfully infection. In addition, the Whatman paper must be kept throughout in close contact with the coleoptile to allow transfer of the conidia to the host tissue. The prepared plates were then returned to the humidity chamber and incubated in the dark for another 48 h. After incubation, the plastic tips were removed from the inoculated coleoptiles. Coleoptiles showing visible symptoms of infection were selected for evaluation under the confocal microscopy. Six coleoptiles per strain were infected. The growth room conditions throughout the experiments were the same as those described for wheat spike infections. The experiment was repeated twice.

### Confocal microscopy

Confocal microscopy was used to explore the expression of fluorescent reporter proteins such as mCherry and GFP in liquid cultures and/or in tissue samples taken from inoculated wheat plants, both floral and coleoptile. Strains expressing GFP under different promoters were inoculated on wheat spikes. At 3 dpi, lemma tissue was isolated using a scalpel blade from spikelets displaying typical symptoms of *Fg* infection. Lemmas were mounted in sterile water into glass slides and GFP emission was observed under confocal microscopy. Protein secretion in wheat coleoptiles were performed with strains expressing either a non-secreted or a secreted version of mCherry. After 2 dpi, the infected epidermis layer was removed from the coleoptile surface in the vicinity of the visible lesion using a surgical blade and mounted onto a glass slide with sterile water. Hyphae tips displaying fluorescence signal were evaluated by confocal microscopy. Germinated spores in liquid media were mounted in TB3 liquid medium onto glass slides. Fluorescence emission from the hyphae was evaluated under the confocal. The wild type PH-1 strain was included in all the evaluations to set the confocal conditions. Fluorescence emission was observed using a Stellaris 8 Falcon confocal microscope (Leica, UK). Excitation/emission wavelengths were 561 nm/590–640 nm and 489 nm/500–530 nm for mCherry and GFP, respectively. The laser intensity was set between 5 and 10% in counting operating mode for both fluorescence signals. Images were analysed using ImageJ and LAS X v3.7 software from Leica. Liquid culture evaluation and wheat spike infections were repeated twice whilst coleoptile infections were done three times. A minimum of three tissue samples / treatment / construct were examined by confocal microscope in each independent experiment.

### RNA extraction and quantitative PCR (qPCR) analysis

Expression of neighbouring genes (FGRAMPH1_01G06815 and FGRAMPH1_01G06817) to the TSI locus 1 and GFP were evaluated by qPCR. Selected transgenic strains as well as the wild type PH-1 strain were grown in YPD or TB3 liquid medium. Briefly, 100 µL of water stocks containing 10^6^ conidia/mL were inoculated in 200 mL of either YPD or TB3 liquid medium. Cultures were grown with agitation (180 rpm) for 36 h at 28 ℃. Mycelia was collected by vacuum infiltration and frozen in liquid nitrogen. Total RNA was extracted using Monarch® Total RNA Miniprep Kit (New England Biolabs, UK). First strand cDNA was synthesised from 1 µg of total RNA using RevertAid First Strand cDNA Synthesis Kit (Thermo Fisher Scientific, UK). The qPCR analyses were performed using Applied Biosystems™ PowerTrack™ SYBR Green Master Mix (Thermo Fisher Scientific, UK) according to the manufacturer’s instructions. Primers efficiencies were calculated by standard curve analysis using six two-fold serial dilutions of pooled cDNA from the *Fg* transgenic strains and PH-1. Primers with 95%-110% amplification efficiencies were used for analysis. Relative gene expression levels were calculated using the method described by Vandesompele et al. [39]. Two housekeeping genes, Actin (FGRAMPH1_01T24551) [40] and histone (FGRAMPH1_01T14929) [41] were simultaneously used for data normalisation. Relative gene expression calculation for the transgenic strains were done in comparison with PH-1. Three independent biological replicates were performed for each fungal strain and treatment. Statistically significant differences in gene expression between *Fg* transgenic lines and PH-1 were calculated using one-way ANOVA followed by Tukey’s post-hoc test.

To test gene expression in wheat floral tissue, selected *Fg* strains were inoculated as previously described. Infected tissues were collected at 4 dpi. Total RNA extraction, cDNA synthesis and gene expression calculations were performed as described above for three independent experiments. Primers used for qPCR are listed in Additional file 2: Table S1.

## Results

### Identification of a conserved micro-region in chromosome 1 suitable for TSI

**Fig. 1:**
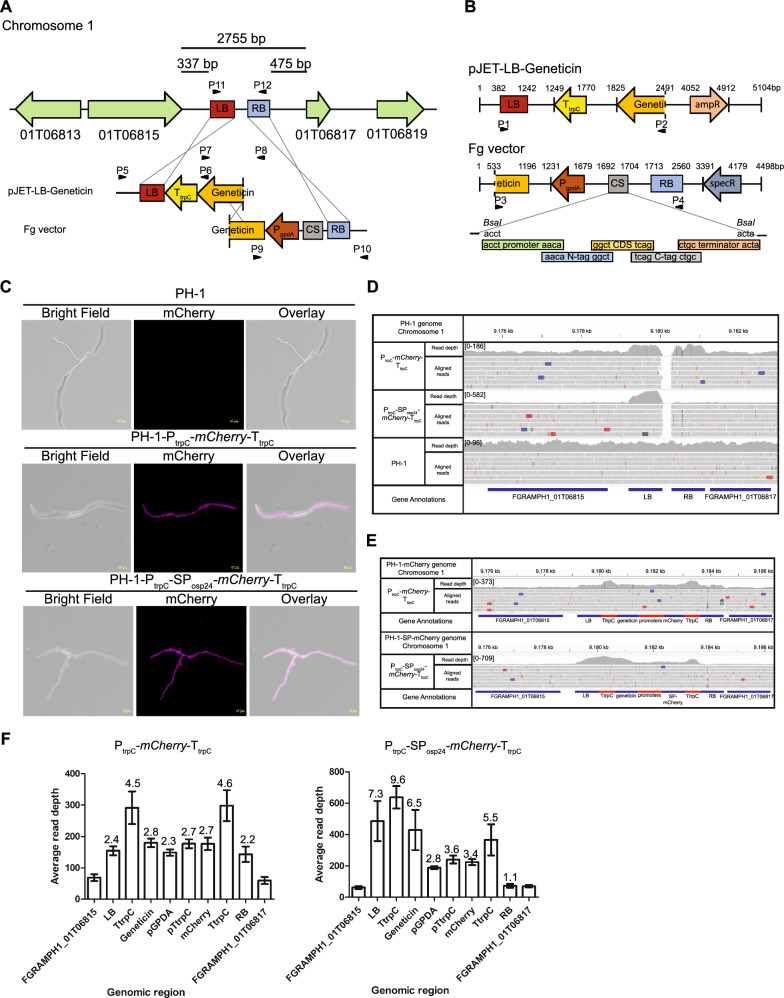
Schematic representation of the locus for TSI and confocal analysis of *Fg* transformants. **A** The TSI locus 1 is located adjacent to a micro-region within chromosome 1 between the genes designated FGRAMPH1_01G06815 and FGRAMPH1_01G06817. At this location, there is an intergenic region of 2.7 kb where the insertion of the expression cassette can occur. To allow integration of the expression cassette in the locus, a vector system was developed based on the split-marker technique. Three recombination events allow insertion of the cassette into the TSI locus 1. Primer combinations P5–P6, P7–P8, P9–P10 and P11–P12 are used to confirm correct cassette integration. **B** A vector system based on the Golden Gate approach was developed to allow cassette integration into the TSI locus 1. PCR fragments amplified by primer combinations P1–P2 and P3–P4 are used for fungal transformation. **C** Confocal images of strains confirming expression of a non-secreted version of mCherry (P_trpC_*-mCherry-*T_trpC_) and a secreted version (P_trpC_*-*SP_OSP24_*-mCherry-*T_trpC_). Fluorescence emissions were detected in 16 h old spore germlings. The wild type strain PH-1 was used as the control to set the confocal conditions. **D** IGV screenshot showing the reads aligned to the LB and RB of the TSI locus 1 in the wild type strain PH-1 and the transformed strains. Numbers in brackets indicate the range of read depth coverage per bp. **E** IGV screenshot displaying reads aligned to the expression cassettes from the transformed strains and the genes flanking the TSI locus 1. **F** Bar graphs represent the average read depth values for each section of the expression cassette and for the two genes (FGRAMPH1_01G06815 and FRGAMPH1_01G06817) flanking the TSI locus 1 for both transformants. Values above the bars are the ratio value calculated as the average read depth value of each section divided by the average read depth value from both flanking genes. Error bars represent standard deviation (SD)

Previously, a conserved micro-region in chromosome 1 suitable for TSI was identified in various *Fusarium* species [13]. In addition, this region was predicted to be in a low recombination region of the *F.* *graminearum* genome [13]. According to Ensembl Fungi (http://fungi.ensembl.org/index.html) [42], there is a wide intergenic region of 3274 bp at the 3′ of the micro-region between the predicted genes FGRAMPH1_01G06815 and FGRAMPH1_01G06817. To confirm that no transcripts had been assigned to this intergenic region, we used publicly available transcriptome data from FungiBD (https://fungidb.org/fungidb/app) [43]. No transcripts were identified inside the intergenic region. However, we identified that the transcript of FGRAMPH1_01T06815 possesses an extra exon at the 3′UTR whilst for FGRAMPH1_01T06817, the transcript prediction from Ensembl Fungi does not match with the transcriptome data from FungiDB. The transcript of FGRAMPH1_01T06817 appears as a single exon. Therefore, the length of the intergenic region is 2755 bp from the stop codon of the FGRAMPH1_01T06815 to the start codon of the FGRAMPH1_01T06817 (Fig. 1A). To permit homologous recombination between the intergenic region and an expression cassette, we decided to use the ‘split-marker’ approach [24]. This approach has been used for gene deletion and complementation in *Fg* [37, 44, 45]. The approach consists in transforming the fungus with two overlapping DNA fragments. Each fragment is flanked by a ~ 1 kb sequence with homology to the target locus. The flanked sequences are defined as left and right borders (LB and RB, respectively). The LB is 337 bp downstream of the stop codon of FGRAMPH1_01T06815 whilst the RB is 475 bp from the start codon of FGRAMPH1_01T06817 (Fig. 1A). Insertion of the expression cassette in the target locus occurs by a triple homologous recombination event. Two events occur between the LB and RB with their respective homologous sequences in the target locus. A third event occurs between the two overlapping DNA fragments (Fig. 1A). We built a vector system to adapt this methodology for target site integration into the 2755 bp intergenic region, now referred to as target site integration locus 1 (TSI locus 1). One vector called pJET-LB-geneticin contains an 816 bp DNA fragment of the *Fg* genome as the LB followed by a resistance cassette. The resistance cassette has the *Aspergillus* *nidulans* *trpC* terminator (T_trpC_) and a 667 bp split fragment of the *geneticin* gene as the selection marker (Fig. 1B). The second vector called the Fg vector contains a 664 bp split fragment of the *geneticin* gene where 536 bp overlaps with the sequence of the geneticin fragment in pJET-LB-geneticin. The 664 bp geneticin fragment is followed by the *A.* *nidulans* constitutive *gpdA* promoter (P_gpdA_) and an 848 bp DNA fragment of the *Fg* genome as the RB (Fig. 1B). Between the promoter P_gpdA_ and the RB, there is a cloning site (CS) adapted to the Golden Gate approach [46] where the type IIS enzyme BsaI cuts twice the vector creating two single-stranded overhangs with 4 bp each. The overhang sequences were defined according to those previously described [19] and thus the modular library developed by the authors can also be used for cloning in the Fg vector (Fig. 1B).

To evaluate if this vector system could or could not be used for transformation of other *Fg* isolates, we aligned the TSI locus 1 sequence with the corresponding genomic regions from other global *Fg* isolates. The TSI locus 1 was found to be conserved among different *Fg* isolates indicating that the vector system can be used to transform strains from different origins. To evaluate if the vector system could also be used for transformation of other Fusarium related species, we performed a blast search using as the query the entire PH-1 TSI locus 1 sequence. Only, four Fusarium species, namely *F.* *asiaticum*, *F.* *culmorum*, *F.* *meridionale* and *F.* *pseudograminearum* showed a high degree of conservation with a 100% query cover and identities within the range 98% to 93% (Additional file 1: Fig. S1C). In *F.* *culmorum* and *F.* *pseudograminearum*, a small insertion of 116 bp in the 5’ of the LB sequence was identified, but we consider that there is enough homology to potentially adapt this strategy for transformation of all four species.

### Transformation into the TSI locus 1

To test if the TSI locus 1 is a suitable region for transformation and gene expression, we built two constructs in the Fg vector. A non-secreted version of mCherry (mCherry) under the control of the constitutive promoter of *A.* *nidulans* *trpC* (P_trpC_) flanked at the 3’ end by the terminator sequence T_trpC_ (P_trpC_*-mCherry-*T_trpC_). A second construct consisted of a secreted version of mCherry (SP-mCherry) where we used the 69 bp secretion signal from *osp24* (SP_osp24_) [22]. The expression of mCherry in this second construct is also controlled by P_trpC_ and has the terminator sequence T_trpC_ (P_trpC_*-*SP_osp24_*-mCherry-*T_trpC_).

**Fig. 2:**
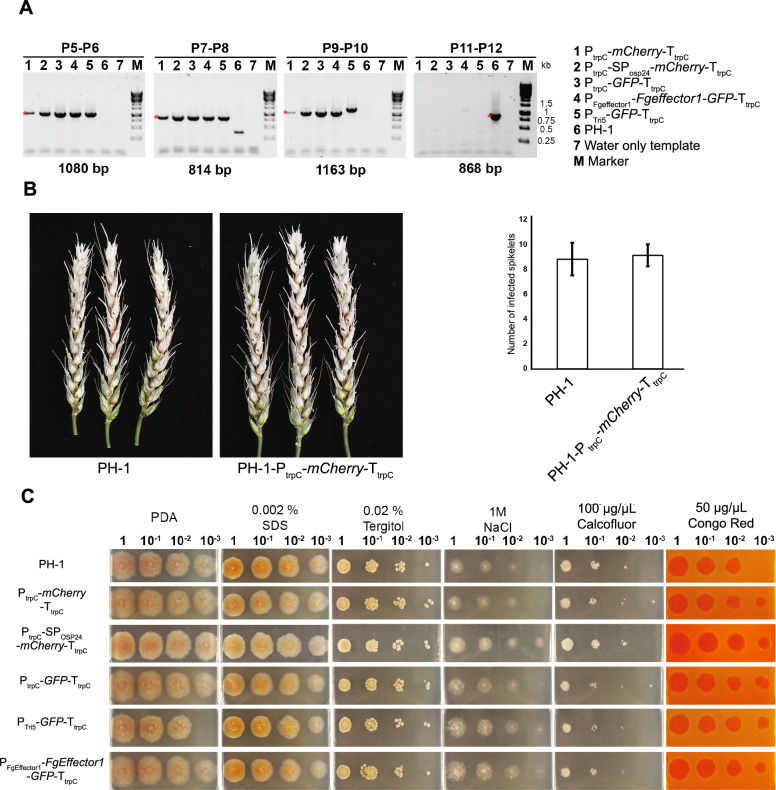
Validation of the transformant strains, floral virulence test and stress evaluations.** A** To select transformants where the cassette was correctly inserted into the TSI locus 1, we amplified four different PCR products. Primer combinations P5–P6 and P9–P10 were used to verify the insertion event. Primer combination P7–P8 evaluated whether the recombination event between the two PCR fragments had been successful. Primer combination P11–P12 tested whether each transformant was homokaryotic for the transgene. Red asterisks indicate the expected PCR size bands. The transformant in lane 5 displayed a slightly higher band size for primer combination P9–P10 when compared to the other transformants. The higher band in lane 5 could be the product of unequal crossover in the RB region of the cassette. **B** Wheat spikes inoculated with PH-1 or the transformant strain (P_trpC_*-mCherry-*T_trpC_). No differences were observed in the number of infected spikelets showing typical disease symptoms. Photographs were taken at 12 dpi. Bars graph shows the number of infected spikelets between PH-1 and the transformant strain. Error bars indicate SD. **C** All the transformed strains showed a similar morphology and growth rate as the wild type PH-1 for all the conditions tested. Photographs were taken after 3 dpi. Salt stress (NaCl), membrane stresses (Calcofluor, Congo Red, Tergitol, SDS). *PDA* potato dextrose agar only

Each cassette was amplified by PCR using the overlapping DNA fragments from pJET-LB-geneticin and the constructs cloned into Fg vector using primer combination P1–P2 and P3–P4, respectively (Fig. 1B). The transformation of the wild type PH-1 strain resulted in ~ 20 geneticin resistant colonies from two 50 ml square plates and six colonies were selected from each transformation event. To validate that the transformations were successful, colonies were evaluated by diagnostic PCR. Primer combinations P5–P6 and P9–P10 were used to confirm that insertion had occurred in the TSI locus 1 (Figs. 1A, 2A). The primer combination P7–P8 was used to confirm that the recombination event between the two overlapping DNA fragments was correct (Figs. 1A, 2A). *Fg* protoplasts might contain more than one nucleus during the transformation and thus not all the nuclei can be transformed during the transformation step [18]. To check that the colonies selected were homokaryotic for the expression cassette, PCR analyses were done on a region between the LB and RB using the primer combination P11–P12. The lack of an 868 bp band belonging to untransformed nuclei showed that all the colonies were homokaryotic (Figs. 1A, 2A). To test for the expression of mCherry, we evaluated mCherry fluorescence emission by confocal microscopy in spores germinated in liquid TB3 media. The colonies selected for each transformation event displayed fluorescence emission (Fig. 1C).

To verify target site integration of the expression cassette in the TSI locus 1 and assess whether deletions might have occurred during transformation, the genomes of the mCherry and SP-mCherry strains were sequenced. The sequencing reads from each strain were aligned to the reference genome of the wild type strain PH-1. Around 99% of the reads from each strain were mapped to the wild type genome to a mean coverage value of 53–66 × for each chromosome (Table 2). For both strains, reads covered the whole genome of PH-1 except for 228 bases between the LB and RB of the TSI locus 1 (Fig. 1D), indicating insertion of the cassette only in the TSI locus 1. Other genomic regions with low read coverage were also detected in the transformant strains (Additional file 3: Fig. S3A). However, these regions were also presence in the aligned sequences obtained from the wild type PH-1 strain. Therefore, we can conclude that there is no evidence of ectopic integration or deletions in both transformants.

**Table 2:** Mapping statistics for the strains sequenced

Transformant strains	Reads after trimming	Reads mapped (%)	Chromosome mean coverage (mean ± sd)			
			Chr1	Chr2	Chr3	Chr4
PH-1	18.205.628	99.73	66.72x ± 11.3	65.70x ± 11.1	66.07x ± 11.1	64.34x ± 16.6
P_trpC_-*mCherry*-T_trpC_	17.665.176	99.70	64.18x ± 11.2	63.57x ± 11.1	63.77x ± 11.1	65.33x ± 14.7
P_trpC_-SP_osp24_-*mCherry*-T_trpC_	15.251.700	99.70	54.97x ± 10.5	54.46x ± 9.9	54.69x ± 9.9	53.81x ± 13.4
PH-1-∆*osp24*-1	13.524.682	99.71	48.37x ± 9.1	48.03x ± 9.1	48.23x ± 9.1	47.04x ± 12.1

To estimate if the cassette was inserted as a single copy in the TSI locus 1, the reads from each strain were aligned to the PH-1 genome containing the sequence of the respective expression cassette inserted in the TSI locus 1, the expression cassettes from both strains were fully covered by reads indicating that both cassettes are complete (Fig. 1E). This evidence agrees with the mCherry expression observed for both strains (Fig. 1C). However, it was observed that the number of reads aligned to the expression cassettes was higher than the number of reads aligned to the two neighbouring genes (FGRAMPH1_01G06815 and FGRAMPH1_01G06817) flanking the TSI locus 1 (Fig. 1F). In the case of the mCherry strain, the ratio values between the average read depth for each section of the cassette and the average read depth for FGRAMPH1_01G06815 and FGRAMPH1_01G06817 genes were 2 to 3 times higher (Fig. 1F). This evidence indicates that together with the insertion of the full cassette, there might be extra copies of the cassette and/or truncations with fragments of the cassette. In the case of the SP-mCherry strain, truncations and/or multiple insertions of the cassette were also observed (Fig. 1E). Ratio values for the regions containing the mCherry gene and the promoters (P_gpdA_ and P_trpC_) were 3 times higher (Fig. 1F). The ratio for the genomic regions containing the LB, the *geneticin* gene and one of the terminators were around 7 times higher in comparison to FGRAMPH1_01G06815 and FGRAMPH1_01G06817 genes (Fig. 1F). Collectively, this evidence indicates that the integration event not only contains a copy of the full cassette, but also truncations containing the LB region, the *geneticin* gene and the *trpC* terminator. For both strains the average read depth for the *TtrpC* terminators was always double the average read depth values from other sections of the cassette (Fig. 1F). The higher values are due to reads that can be aligned to both terminator sequences.

The higher number of reads aligned to different sections of the cassette indicates the existence of truncations and/or tandem insertion of the cassette. To identify truncations and/or tandem insertion of the cassette, unmapped reads were recovered after alignment with the PH-1 genome. A de novo assembly approach was performed using the unmapped reads to identify contigs with evidence of truncations and/or tandem insertions. In the case of the mCherry strain, a contig of 263 bp (Contig_42) containing truncated sequences of the RB (RB_727-852_) and LB (LB_1-117_) was identified (Additional file 3: Fig. S3B). In the SP-mCherry strain three contigs were identified. A contig of 366 bp (Contig_11) containing truncated sequences of the LB (LB_248-130_) and RB (RB_131-383_). A second contig of 396 bp (Contig_9) was identified containing truncated sequences of the LB (LB_723-861_ and LB_1-61_) and *trpC* terminator (*TrpC*_1-180_). Finally, a third contig of 200 bp (Contig_15) containing truncated sequences of the *geneticin* gene (Gen_204-127_) and the LB (LB_1-118_) was also identified (Additional file 3: Fig. S3B). These datasets indicate the existence of fragments containing truncated sequences from the LB, *geneticin* gene and *trpC* terminator and thus explain the higher number of read mapped onto that region of the cassette.

The data from Contig_42 in the mCherry strain and Contig_11 in the SP-mCherry strain may also indicate the existence of head-to-tail tandem insertion of the cassette. To test for the existence of tandem insertion of the cassette, we evaluate by PCR the insertion length. A single insertion of the mCherry cassette in the mCherry strain has a length of 5471 bp spanning from primer P5 to primer P10 which anneal outside the TSI locus 1 (Fig. 1A). In case of a tandem insertion, the expected size of the PCR product in this transgenic line should be 10,768 bp, a length suitable for amplification by PCR (Additional file 3: Fig. S3C). Amplification using the primer combination P5-P10 generated a PCR product of around 8 kb (Additional file 3: Fig. S3C). This 8 kb PCR product indicates the insertion of a full copy of the cassette at the TSI locus 1 as well as a truncated copy containing fragments of the cassette.

Potential structural variants indicated by coloured reads in the IGV screenshots were observed in the TSI locus 1 region and in chromosomic regions with low read coverages such as telomeres (Fig. 1D, E; Additional files 3 and 4: Figs. S3A, S4C, D). This included deletions (red), insertions (blue), inversion (gray-blue) and duplications or translocations (green) [32]. In the case of the TSI locus 1 region, coloured reads were minimal and randomly distributed. Therefore, we assume that these are not true variants. In the case of regions with low coverage, coloured reads were present not only in the transformed strains but also in the wild type strain (Additional file 3 and 4: Fig. S3A, S4D). Hence, coloured reads could be the consequence of poor coverage and/or differences with the reference genome used to align the reads.

### Insertion in the TSI locus 1 does not affect fungal infection

To test if integrations in the TSI locus 1 could affect fungal infection and disease symptom causing ability, a floral point inoculation test was done. Wheat spikes of the susceptible cv Bobwhite were inoculated with either wild type untransformed PH-1 and the strain expressing the non-secreted version of mCherry. There were no differences in the number of infected spikelets between PH-1 and the mCherry strain indicating that integration in the TSI locus 1 does not affect the virulence of the fungus (Fig. 2B). We also test if integrations in the TSI locus 1 affected the fungal morphology or growth rate when the fungus was grown in vitro under normal or different stress conditions. PH-1 and the transformants were grown in PDA plates containing either salt or membrane stresses. The transformants showed a similar morphology and growth rate as PH-1 for all the conditions tested (Fig. 2C). Hence, insertions in the TSI locus 1 does not affect the fungal growth under the conditions tested.

### Validation of protein secretion in *F. graminearum*

**Fig. 3:**
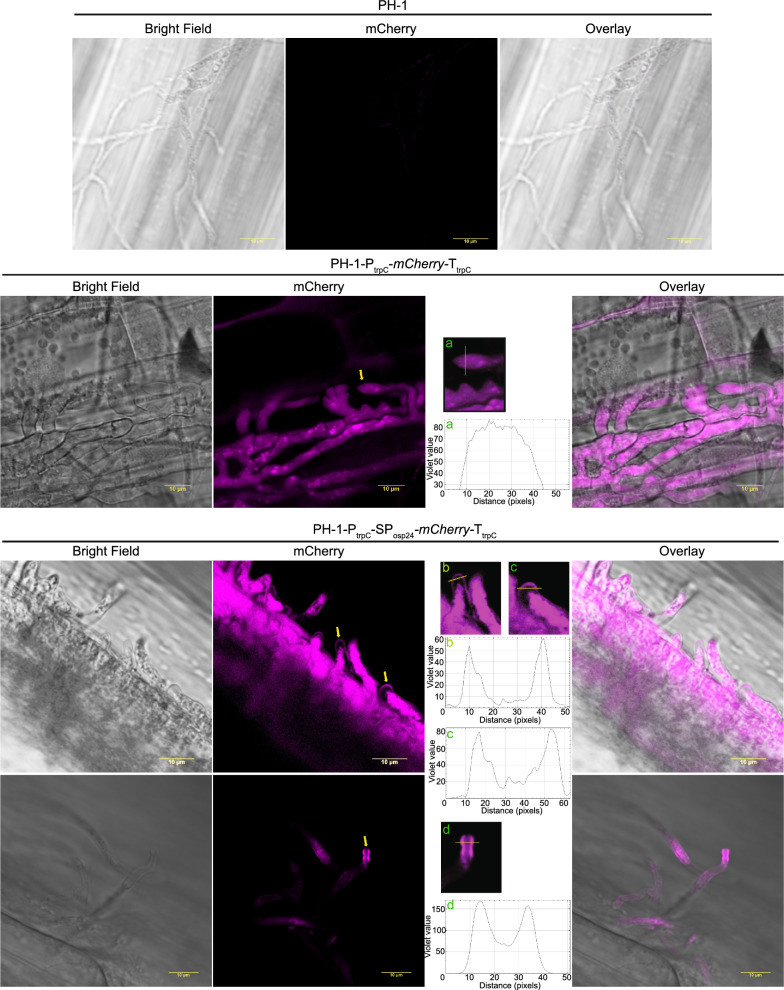
Secretion of mCherry in wheat coleoptiles. Confocal images of wheat coleoptiles infected with PH-1 or the strains expressing either the non-secreted version of mCherry (P_trpC_*-mCherry-*T_trpC_) or the secreted version (P_trpC_*-*SP_osp24_*-mCherry-*T_trpC_). The strain expressing the non-secreted version displayed accumulation of the mCherry fluorescence signal solely inside the hyphae. Whereas the strain expressing the secreted version displayed accumulation of the fluorescence signal in the periphery mainly localised towards the tips. Graphs a, b, c and d indicate mCherry signal intensity determined along the diameter of the hyphae, taken at the positions indicated by yellow lines in each image enlarged and labelled with the same letter. The yellow arrows point to the selected hyphae used for this analysis. Wild type strain PH-1 was used to set confocal conditions. Confocal images were taken 2 dpi

The yeast secretion trap assay is a commonly used method to validate protein secretion [47]. However, in plant pathogens such as the fungus *U.* *maydis* and *M.* *oryzae*, secretion studies are performed in the native system. A protein predicted to be secreted is fused to a fluorescence reporter and the fungus is transformed with the recombinant protein. If the protein is secreted, an accumulation of the fluorescence signal can be observed in the periphery of the infected hyphae whilst a non-secreted protein will only accumulate inside the hyphae [48–50]. We evaluate whether the *Fg* strains expressing the secreted and non-secreted versions of mCherry show similar or dissimilar distribution pattern. We infected wheat coleoptiles with the non-secreted version of mCherry (P_trpC_*-mCherry-*T_trpC_) and the secreted version (P_trpC_*-*SP_osp24_*-mCherry-*T_trpC_). The strain expressing the non-secreted version displayed accumulation of the mCherry fluorescence signal inside the hyphae. However, the strain expressing the secreted version displayed accumulation of the fluorescence signal in the periphery of the hyphae mainly localised towards the tips (Fig. 3). These results indicate that secretion studies can also be performed in *Fg* using cassettes expressed from the TSI 1 locus.

### Complementation of PH-1-Δ*osp24* by insertion into the TSI locus 1 restores full virulence

**Fig. 4:**
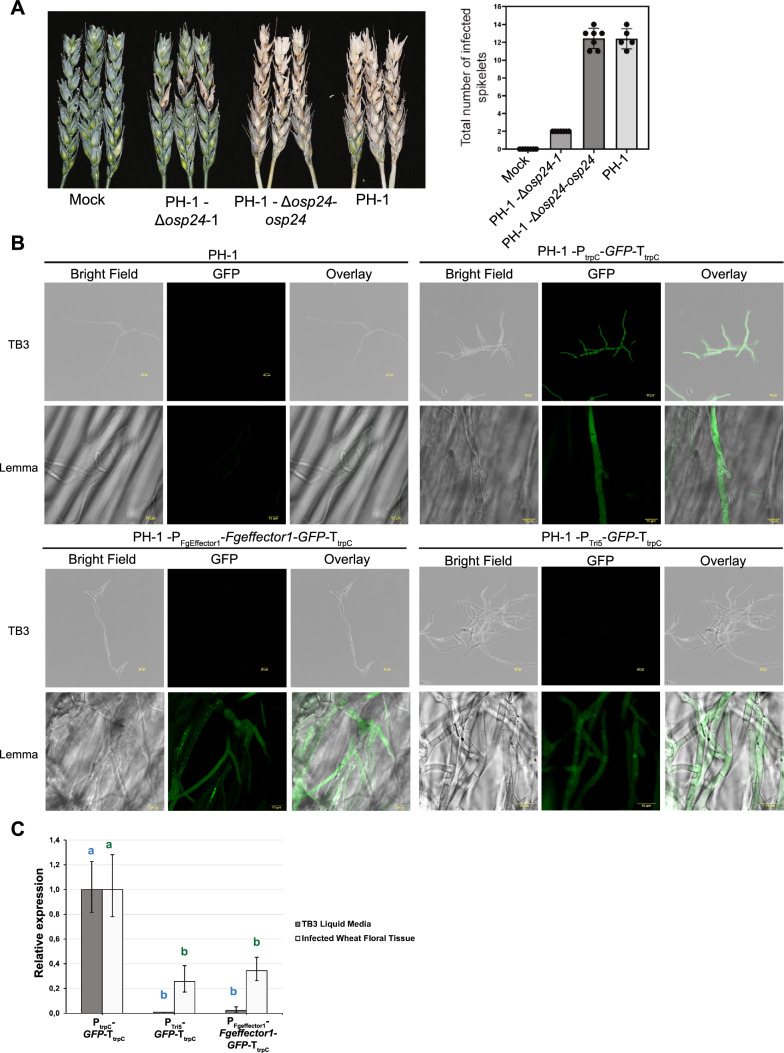
Complementation of PH-1-*Δosp24-1* and promoter analyses under different conditions.** A** Complementation of the PH-1-Δ*osp24*-1 mutant strain with the *osp24* gene residing within the TSI locus 1 (PH-1-Δo*sp24*-*osp24*-1) restores full virulence. Photographs were taken at 14 dpi. Marked spikelets in each floral spike indicate the inoculation points. Bar graph shows no differences in the number of infected spikelets between PH-1 and PH-1-Δ*osp24*-1-*osp24* whilst the mutant strain shows reduced virulence. Visibly diseased spikelets were counted after 14 dpi and include the point of inoculation. Mock indicates plants inoculated with water. Error bars indicate SD. **B** Confocal images of strains expressing GFP under the control of different promoters. Strain expressing constitutive GFP (P_trpC_-*GFP*-T_trpC_) displayed fluorescence when *Fg* was grown in TB3 liquid medium as well as during wheat spike infection (lemma). Expression of GFP under the control of the trichodiene synthase promoter (P_Tri5_-*GFP*-T_trpC_) or an effector promoter (P_Fgeffector1_-*FgEffector1*-*GFP*-T_trpC_) only occurs during infection. The PH-1 strain was used as control to set confocal conditions. Images were taken at 3 dpi. **C** Relative expression of GFP for strains expressing GFP under the control of the promoters P_Tri5_, P_Fgeffector1_ and P_trpC_ during growth in TB3 and wheat spike infection. Data represent mean of three replicates. Error bars denote the 95% confidence interval. Statistically significant differences between P_trpC_-*GFP*-T_trpC_ with P_Tri5_-*GFP*-T_trpC_ and P_Fgeffector1_-*Fgeffector1*-*GFP*-T_trpC_ were calculated using one-way ANOVA followed by Tukey post-hoc test (*P* < 0.05)

Previous studies have shown that o*sp24* (FGRAMPH1_01G15939) is required for successful infection and disease formation within wheat floral tissue [22]. Therefore, the o*sp24* gene was a good candidate to test if the TSI locus 1 can be used for complementation analysis. The o*sp24* gene was mutated in PH-1 by the split marker approach. Analysis by PCR confirmed that the coding sequence of o*sp24* was replaced by the hygromycin cassette (Additional file 4: Fig S4A). Further, sequencing analysis of the mutant strain (PH-1-Δ*osp24*-1) showed a 480 bases gap in the coding sequence of *osp24* (Additional file 4: Fig. S4C). The Hyg cassette was integrated in the *osp24* locus (Additional file 4: Fig. S4C). In addition, there was no evidence of ectopic integration as genomic regions with low coverage in PH-1-Δ*osp24*-1 were also present in PH-1 (Additional file 4: Fig. S4D). Finally, the Hyg cassette was inserted as a single copy as the ratio values between the different sections of the cassette (LB, hygromycin cassette, and RB) and the genes (FRGRAMPH1_01G15937 and FGRAMPH1_01G15941) flanking the *osp24* locus were close to 1 (Additional file 4: Fig. S4C). *In* *planta* testing of this mutant strain in Apogee wheat spikes showed reduced virulence compared to the wild type PH-1 strain (Fig. 4A). The infection was limited to the inoculated spikelets as previously reported [22]. To test for the full restoration of virulence, PH-1-Δ*osp24*-1 mutant strain was complemented by inserting the expression cassette (P_osp24_*-osp24-*T_osp24_) containing the wildtype gene into the TSI locus 1. The complemented strains were selected by PCR to confirm correct insertion of the P_osp24_*-osp24-*T_osp24_ cassette into the TSI locus 1 (Additional file 4: Fig. S4B). The complemented strain PH-1-Δ*osp24*-*osp24*-1 regained the wildtype virulence phenotype (Fig. 4A). In addition, we tested if the Δ*osp24* mutant strain as well as the complemented strain were affected in fungal morphology or growth rate. The two transgenic strains together with PH-1 were grown in vitro under different stress conditions. The mutant as well as the complemented strain showed a similar morphology and growth rate as PH-1 for all the conditions tested. Therefore, mutation in the *osp24* locus affects virulence but not the *Fg* growth rate under the stress conditions tested (Additional file 5). These results indicate that the TSI locus 1 can be used for efficient gene complementation studies.

### Virulence specific promoter activity is not altered by insertion into the TSI locus 1

Pathogens possess genes that are exclusively expressed during host infection. *Fg* produces different trichothecene mycotoxins that are required for successful infection of wheat spikes. The *Tri5* gene codes for an enzyme that catalyses the first step in the production of all trichothecene mycotoxins [51, 52]. The *Tri5* gene is highly expressed during infection [53], but only a low expression level is observed in liquid culture unless transferred to specific induction media [54] or by the addition for specific inducers to the cultures for example agmatine [55] or hydrogen peroxide treatments [56]. To test if native promoter activity might be affected by insertion in the TSI locus 1, we cloned in the Fg vector, a construct where GFP expression was under the control of the *Tri5* promoter (P_Tri5_-*GFP*-T_trpC_). In addition, we cloned a promoter from a candidate effector gene FgramPH1_01G11655 (P_Fgeffector1_) that according to transcriptomic analysis is upregulated during the symptomatic phase of the wheat spike infection [57]. A second construct was built where the P_FgEffector1_ promoter controlled the expression of the *FgEffector1* gene which was also C-terminally tagged to GFP (P_Fgeffector1_-*Fgeffector1*-*GFP*-T_trpC_). Finally, as an additional control a construct was generated where GFP was under the control of the constitutive promoter P_trpC_ (P_trpC_-*GFP*-T_trpC_). Positive transformants were obtained for the three different constructs (Fig. 2A). In addition, the transformant strains showed a similar morphology and growth rate as PH-1 for all the nutrient and stress conditions tested (Fig. 2C). To test promoter specificity, selected strains for the three different constructs were grown in TB3 liquid medium. Only the strain expressing constitutive GFP displayed a fluorescence signal. However, when the same strains were used to infect wheat spikes, all the strains displayed fluorescence (Fig. 4B). To confirm that the GFP fluorescence signal observed among the strains was due to GFP mRNA expression levels, we performed qPCR analyses. When the strains were grown in TB3 liquid medium, only the strain expressing constitutive GFP showed expression. The strains expressing GFP under the control of either the P_Tri5_ or P_FgEffector1_ promoters showed relative expression values close to zero in comparison to the strain expressing constitutive GFP (Fig. 4C). When the strains were used to infect wheat spikes, all three strains expressed GFP. The strains expressing GFP controlled by either the P_Tri5_ or P_FgEffector1_ promoters displayed relative expression values of 0.26 and 0.34, respectively in comparison to the strain expressing constitutive GFP (Fig. 4C). The lower expression values in these two lines were expected because the P_Tri5_ and P_Fgeffector1_ are virulence specific promoters whilst the P_trpC_ is a constitutive promoter. Finally, the differences observed between the strains P_Tri5_-*GFP*-T_trpC_ and P_Fgeffector1_-*Fgeffector1*-*GFP*-T_trpC_ and the P_trpC_-*GFP*-T_trpC_ strain were significantly different for both treatments (Fig. 4C). Hence, the qPCR results validate the GFP fluorescence emission observed in the confocal analyses and indicate that the activities of the two promoters P_Tri5_ and P_Fgeffector1_ were not altered by their location in the TSI locus 1.

### Integration into the TSI locus 1 does not affect the expression of the flanking genes

**Fig. 5:**
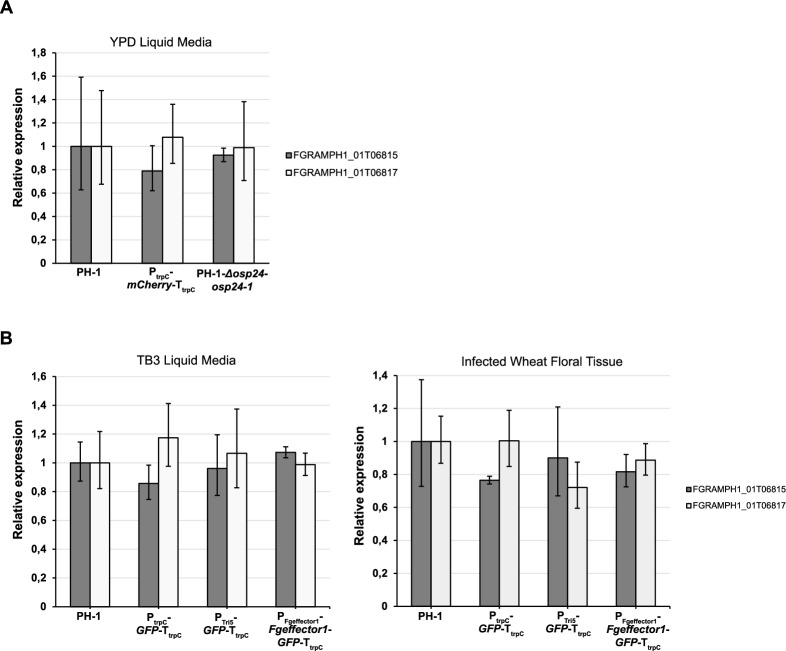
qPCR analysis of expression levels for the genes flanking the TSI locus 1.** A–C** Relative expression for the genes FGRAMPH1_01T06815 and FRGAMPH1_01T06817 in different *Fg* transgenic strains during growth in YPD, TB3 and following wheat infection, respectively. Data represents the mean of three replicates. Error bars denote the 95% confidence interval. No statistically significant difference between PH-1 and the *Fg* transgenic strains were found (one-way ANOVA followed by Tukey post-hoc test, *P* < 0.05)

To test if integrations affect the expression of the flanking genes to the TSI locus 1, we designed qPCR primers for both genes (FGRAMPH1_01G06815 and FGRAMPH1_01G06817) taking into consideration the transcriptomic data from FungiDB. Both genes were known to be expressed in YDP medium as well as in infected wheat floral tissue according to publicly available transcriptomic data [57]. We explored the mRNA expression levels of these two genes by qPCR in the strains expressing the non-secreted version of mCherry (P_trpC_*-mCherry-*T_trpC_), the complemented strain PH-1-Δ*osp24*-*osp24*-1 and the wild type strain PH-1. The relative expression values for both genes in the mCherry and complemented strains were close to 1 as observed in PH-1 (Fig. 5A). The mCherry strain displayed a relative expression value of 0.8 for the FGRAMPH1_01T06815 transcript in comparison with PH-1, indicating a subtle reduction in gene expression. However, the statistical analysis showed no significantly differences in the expression levels for both genes between the transgenic strains and PH-1 (Fig. 5A). We also tested the expression of FGRAMPH1_01T06815 and FGRAMPH1_01T06817 in the transgenic strains expressing P_Tri5_-*GFP*-T_trpC_, P_Fgeffector1_-*Fgeffector1*-*GFP*-T_trpC_, P_trpC_-*GFP*-T_trpC_ and PH-1 in TB3 medium and in infected wheat floral tissue. No significant differences were observed between the transgenic strains and PH-1 either when grown in TB3 or during wheat infection (Fig. 5B). Therefore, integrations into the TSI locus 1 do not alter the expression of the flanking genes.

## Discussion

*F.* *graminearum* is an important disease-causing pathogen worldwide that impacts on global food and feed security. The functional characterisation of genes and proteins in this pathogen is required in order to develop disease control strategies. Common approaches to study gene/protein functions include gene complementation and protein localisation. These approaches include the integration of expression cassettes into the fungal genome, but frequently these integration events are non-targeted and therefore occur randomly. The identification of a suitable locus for TSI in *Fg* is required to avoid the potential risks of ectopic integration. Insertion of a cassette elsewhere in the genome can alter the functions of the genes flanking the insertion site. In addition, the cassette can be inserted inside a gene locus. Loss and/or changes to gene expression may not have a direct effect on the phenotype under study. However, the presence of ectopic integration events could be relevant when transcriptomic and/or metabolomics analysis are also envisaged. Another advantage of TSI is that this approach permits a direct comparison of promoter activities or gene functions within different genetic backgrounds.

In this study, the TSI locus 1 located within chromosome 1 was shown to be a suitable region for cassette insertion; we observed good levels of expression for six different cassettes. We observed that the *in* *planta* specific expression of three promoters (P_osp24_, P_Tri5_ and P_Fgeffector1_*)* was not affected by insertion in the TSI locus 1. We did not observe phenotypic differences between the transformed strains and the wild type strain for the different stresses evaluated and during wheat infection. Full virulence was restored when the *osp24* mutant was complemented with a full copy of the *osp24* gene targeted to TSI locus 1. Finally, cassette insertions did not affect the expression of the genes flanking the TSI locus 1.

The expression of the two genes flanking the TSI locus 1 (FGRAMPH1_01G06815 and FGRAMPH1_01G06817) was tested by qPCR in five out of six *Fg* transgenic strains generated in this study. We only observed a subtle reduction in the FGRAMPH1_01T06815 expression for the P_trpC_-*mCherry*-T_trpC_ and P_trpC_-GFP-T_trpC_ lines. However, we did not observe statistically significant differences in gene expression for any of the transgenic strains in comparison with PH-1. According to Uniprot (https://www.uniprot.org/) [58], FGRAMPH1_01G06815 is predicted to be a member of the Spc97/Spc98 family of spindle pole body (SBP) component. Members of this protein family are involved in microtubule formation [59]. If there is a subtle downregulation in the mCherry and GFP lines, both lines appear not to be affected. Wheat infection with the mCherry line exhibited a virulence phenotype indistinguishable from wild type. Both strains showed similar growth rate and morphology in comparison with PH-1 under the different stress conditions tested in vitro.

The TSI locus 1 can be used for the transformation of different *Fg* strains due to the high levels of sequence conservation. In addition, the locus is highly conserved in four FHB disease causing Fusarium species (*F.* *asiaticum*, *F.* *meridionale*, *F.* *culmorum* and *F.* *pseudograminearum*) that belong to the same *Fusarium* Species Complex [60]. This evidence indicates that the Golden Gate based vector system can potentially be used in these additional Fusarium species. However, this hypothesis must be formally tested by transformation of each Fusarium species with the vector system.

Whole genome sequencing analysis showed insertion of the expression cassette only in the TSI locus 1 without any evidence of ectopic integration elsewhere in the genome. In addition, the expression cassettes from the mCherry and SP-mCherry strains were shown to be completed with full read coverage and mCherry expression observed in both strains. The bioinformatic analysis showed some contigs containing evidence of truncated sequences of the cassette and Contig_42 and Contig_11 may also indicate the existence of tandem insertion in the mCherry and SP-mCherry strains, respectively. PCR amplification of the insert from the mCherry strain showed an amplicon of around 8 kb rather than the predicted 10 kb for a tandem insertion of the cassette. A PCR product length of 8 kb indicates the existence of a full copy of the cassette plus a truncation of the cassette to around 2.6 kb. Long-read sequencing approaches such as PacBio or Nanopore would enable a better characterisation of the insertion events because the short-read (150 bp) sequencing approach used in this study does not provide enough resolution. Our aim was to obtain transformed strains that express sufficient fluorescent signal to be detected during plant infections. We can conclude that the bespoke bioinformatic pipeline allowed us to rule out the existence of ectopic integrations, deletions elsewhere in the genome, verified the insertion of the expression cassette in the TSI locus 1 and predicted the existence of cassette truncations. Finally, Southern blot analysis is the gold standard technique used to test for ectopic integration and predict number of copies inserted during transformation [61–63]. However, the technique cannot detect structural variants such as deletions occurring elsewhere in the genome during the transformation process. Therefore, the bioinformatic approach together with PCR amplification of the insert could serve as an alternative to Southern blotting analysis.

The existence of truncations per se are not related with integrations in the TSI locus 1 because complementation of the *osp24* mutant strain in the TSI locus 1 showed a single insertion of the cassette. It is noteworthy that multiple tandem insertion of the cassette during transformation has been reported in other fungi where the selection marker could be responsible for the concatenation [63–65]. The existence of truncations in the mCherry and SP-mCherry strains could be a consequence of the prior screening criteria chosen, namely transgenic strains were first selected by scoring for the intensity of the fluorescence signal.

Currently, the well-established yeast recombinational cloning approach seems to be one of the preferred options for gene fusions in different *Fg* studies [7–9]. The methodology exploits yeast homologous recombination to fuse a gene with different promoters or tags [66]. However, this cloning strategy is somewhat tedious because the first cloning step has to be done in yeast. Then, the plasmid is purified, transformed into *E.* *coli* and then*,* amplified to permit the cloned product to be sequenced. The Golden Gate based vector system developed allows the assembly of a gene of interest with different tags and/or promoters in a restriction-ligation reaction without the necessity of using yeast. In addition, the methodology reduces the overall cloning time and allows the use of a library of modules already available. We have not tested the maximum length of the expression cassette that can be inserted into the TSI locus 1. However, in our hands the protocol was highly efficient for all the constructs tested.

Pathogens secrete many different types of proteins during infections. Candidate secreted proteins are usually identified bioinformatically by the predicted presence of a secreted signal at the N terminus [67]. Validation of protein secretion is commonly assessed by using the yeast secretion trap assay. The approach consists of fusing the cDNA of a potential secreted protein to the yeast invertase (suc2) reporter gene lacking its signal peptide. If the protein is secreted, this extracellular targeting allows the growth of a yeast strain defective in suc2 on a sucrose selection media [47]. In *Fg*, validation of protein secretion is often done using the yeast secretion trap assay [9, 22, 68]. Even though the technique is widely accepted, these heterologous results should ideally be verified with experiments performed in the native system. Secretion studies have been developed for different pathogens such as *U.* *maydis* and *M.* *oryzae* [48–50]. We found that *Fg* hyphae tips harbouring the secreted mCherry version displayed accumulation of the fluorescence signal in the periphery whilst the non-secreted strain accumulated the fluorescence signal inside the hyphae. These comparative results indicate that the technique might also be exploitable for *Fg*. The technique was performed in wheat coleoptiles; a tissue easy to manipulate and visualise under confocal microscopy. However, not all *Fg* genes may display a similar expression pattern among different host tissues [69]. Therefore, the future identification of *Fg* promoters highly induced during coleoptile infection would be required to apply this technique successfully. Finally, a major challenge faced during the setting-up of the technique was the infection strategy per se of *Fg*. Both, *U.* *maydis* and *M.* *oryzae* infect single cells at earlier time points of infection, allowing the easy identification of hyphae tips and strong fluorescence signal accumulation around the hyphae [70, 71]. However, *Fg* grows very fast throughout the infected tissue producing various types of infective hyphae and structures making it difficult to find hyphal tips with strong fluorescence signals. In our studies, the best results were obtained 48 h post infection in colonised areas where hyphae were growing exclusively intracellularly.

## Conclusion

In this study, we characterised and functionally tested a locus for TSI in *F.* *graminearum,* the first described for this pathogen. Cassette insertion into the TSI locus 1 does not affect fungal virulence and growth under different stress conditions. We observed good levels of expression for all the expression cassettes tested. In addition, promoter activities were not affected by insertion in the TSI locus 1. The high degree of sequence conservation of the locus would allow the transformation of different *Fg* isolates and potentially some other related phytopathogenic Fusarium species. We developed a vector system for efficient cloning and transformation into the TSI locus 1. We designed a bespoke bioinformatic pipeline that together with PCR amplification of the insert could be used as an alternative to Southern blotting analysis. Finally, we established a protocol for protein secretion studies using confocal microscopy and tested the suitability of the TSI locus 1 for stable expression of different gene fusions. Hence, the TSI locus 1 and the new modular Fg vector system are versatile tools to study gene/protein functions in *F.* *graminearum*.

### Supplementary Information

**Additional file 1:** Cloning steps to build Fg vector and pJET-LB-Geneticin vectors, and LB and RB sequence alignments.** A)** A geneticin resistance cassette was built using the Golden Gate cloning approach followed by PCR to amplify a product containing the *gpdA* promoter and a fragment of the *geneticin* gene (P_gpdA_*-geneticin*_1-664_). Next, the RB of the TSI locus 1 was amplified from PH-1 genomic DNA. The PCR products from the RB and P_gpdA_*-geneticin*_1-664_ were digested and ligated. During the ligation process a Golden Gate cloning site (CS) was created. A fragment containing a spectinomycin resistance cassette (specR) and a bacterial origin of replication (ori) was amplified by PCR from the pGreen vector. Finally, the specR-ori and the *geneticin*_1-664_-P_gpdA_-CS-RB PCR products were digested and ligated to build the Fg vector. **B)** From the geneticin cassette a PCR product was amplified containing a fragment of the *geneticin* gene fused to the T_trpC_ terminator (T_trpC_-*geneticin*_128-795_). The LB of the TSI locus 1 was amplified by PCR from PH-1 genomic DNA. The PCR products containing the LB and the T_trpC_-*geneticin*_128-795_ were digested and ligated. The ligation product was amplified by PCR and cloned into pJET. **C)** DNA sequence alignments of the LB and RB sequences from PH-1, various other *Fg* isolates and other Fusarium species. Asterisks indicate positions which have a conserved base among all the isolates and species. *F.* *graminearum* (F. gram) isolates: PH-1 (US, GenBank-accession: PRJNA13839), GZ3639 (US, GenBank-accession: PRJNA19849); MDC_Fg1 (France, GenBank-accession: UIHA00000000.2); CS3005 (Australia, GenBank-accession: PRJNA235346); CML3066 (Brazil, GenBank-accession: PRJEB12819) and Fg-12 (China, GenBank-accession: PRJNA743144). *F.* *culmorum* (F. culm) UK99 (UK, GenBank-accession: PRJEB12835), *F.* *pseudograminearum* (F. pseu) Fp22-2F (China, GenBank-accession: PRJNA871792). *F.* *meridionale* (F. meri) JX18-4 (China, GenBank-accession: PRJNA977470) and *F.* *asiaticum* (F. asia) KCTC_16664 (South Korea, GenBank-accession: PRJNA784645).

**Additional file 2: Table S1. **List of primers used in this work. Primers are listed in order of appearance.

**Additional file 3:** Genomic sequence analysis of the mCherry expressing strains and PCR amplification of the insertion.** A**) IGV screenshots displaying genomic regions with low read depth coverage. Regions with low read depth were identified not only in the transformant strains but also in PH-1. Low coverage regions were usually identified at the telomeres (Chr1I, Chr1IV, Chr2, Chr3I, Chr3II and Chr4IV), in the 5’UTRs (Chr1II, Chr1III and Chr4I) or 3’UTRs (Chr4II and Chr4III) of different genes. The red bar above the figure indicates the chromosomic region with read depth values ≤ 1. **B**) Contigs with evidence of cassette truncations and/or tandem insertions for the mCherry and SP-mCherry strains. Graphs above the contigs represent the read depth for each base of the contig. **C**) PCR amplification of the insertion at the TSI locus 1 in the mCherry strain. Graph represents the predicted tandem insertion for the mCherry line.

**Additional file 4: **Genotyping and genomic analysis of PH-1, PH-1-Δ*osp24* mutant and the Δ*osp24* complemented strains.** A)** To select strains where the *osp24* gene was deleted, three different PCR products were amplified. Primer combinations O11–O12 and O13–O14 were used to verify the insertion of the Hyg cassette into the *osp24* locus. Primer combination O9–O10 evaluates whether the *osp24* coding sequence was deleted. **B)** To select PH-1-Δ*osp24* complemented strains, five different PCR products were amplified. Primer combinations P5–P6 and O9 and P10 were used to test for correct insertion of the cassette into the TSI locus 1. Primer combinations P7–P8 and P11–P12 were used to evaluated successful recombination between the two PCR fragments and whether each transformant was homokaryotic for the transgene, respectively. Primer combination O9–O10 evaluates the presence of *osp24* in the transformed strains. Red asterisks indicate the expected PCR size bands. **C)** Upper IGV screenshot shows sequencing reads aligned to the FGRAMPH1_01G15939 (*osp24*) genomic region in PH-1 and PH-1-Δ*osp24*-1. Lower IGV screenshot shows that the coding sequence of *osp24* was replaced by the hygromycin cassette. Bar graph (right) represents the average read depth values for the hygromycin cassette and the two genes (FGRAMPH1_01G15937 and FGRAMPH1_01G15941) flanking the *osp24* locus. Values above the bars are the ratio value calculated as indicated above. Error bars represent SD of each average coverage value. **D)** IGV screenshots displaying genomic regions with low read coverage in the PH-1-Δ*osp24* mutant strain and PH-1. Regions with low coverage were usually identified at the telomeric regions (Chr1I, Chr1IV, Chr2, Chr3II). Other regions such as 5’UTRs (Chr1II, Chr1III and Chr4I) or 3’UTRs (Chr4II) of different genes and an intergenic region (Chr3I) were identified. The red bar above the figure indicates the chromosomic region with read coverage values ≤ 1. Values shown in brackets in the coverage section are the count range for the bar graph.

**Additional file 5: **Stress tests for PH-1, Δ*osp24* mutant strain and *osp24* complemented strain. The mutant strain as well as the complemented strain showed a similar morphology and growth rate as PH-1 for all the conditions tested. Photographs were taken after 3 dpi. Salt stress (NaCl), membrane stresses (Calcofluor, Congo Red, Tergitol, SDS). PDA: potato dextrose agar only.

## Data Availability

The data supporting all the findings of this study are available within the paper and its supplementary data. Raw read data from the different sequenced strains are available at ENA (European Nucleotide Archive) with accession number # PRJEB64490.
