# Supplementary material for: Identification and functional characterisation of a locus for target site integration in Fusarium graminearum
Source: Fungal Biol Biotechnol. 2024 Feb 26;11:2. doi: 10.1186/s40694-024-00171-8 (PMC10898126; doi:10.1186/s40694-024-00171-8)
Supplement: Supplementary file 2 — Additional file 2: Table S1. List of primers used in this work. Primers are listed in order of appearance. [file 40694_2024_171_MOESM2_ESM.pdf]

Table S1. List of primers used in this work

Primer	5’-3’ sequence	Fragment amplified
Dest_F SapI	TATGCTCTTCATCAGGAGATCTCATGTGAG	Spec-Ori from pGreen
Dest_R SapI	TATGCTCTTCATGTTTAATTCCGGGGATCG	
FgRB_R SapI (P4)[2]	TATGCTCTTCATGACCAGAACCACCAATAACTG	RB from PH-1
FgRB_F XhoI	TATCTCGAGGGTCTCTACTAAGACCCAAGGACAGGTTGC	
pGPDApro_F	ATATGGTCTCAACCTCCTCACTCCACCATGTTGG	*gpda* promoter
pGPDApro_R	ATATGGTCTCATGTTTTTGAAGATTGGGTTCCTC	
TtrpC_F	ATATGGTCTCACTGCCACTTAACGTTACTGAAATC	*trpC* terminator
TtrpC_R	ATATGGTCTCATAGTGGTACCAGTTCAAGGAAGAAACAGTGC	
Gene_F1 (P7)[2]	ATATGGTCTCAGGCTGTATGATCGAGCAGGACGGACTCC	*Geneticin* gene
Gene_R1 (P8)[2]	ATATGGTCTCACTGATCAGAAGAATTCGTCCAACAGG	
Gene_R XhoI	TATCTCGAGGGTCTCAAGGTCCTCACTCCACCATGTTGG	geneticin$_{1\text{-}664}$-P$_{gpdA}$
Gene_F BsaI	ATAGGTCTCACGATGTCCTGGTAGCGATCC	
Gene_F SapI (P3)[2]	TATGCTCTTCAACACGATGTCCTGGTAGCGATCC	geneticin$_{1\text{-}664}$-P$_{gpdA}$-RB
FgRB_R SapI (P4)[2]	TATGCTCTTCATGACCAGAACCACCAATAACTG	
TtrpC_F AgeI	ATATACCGGTGGTACCAGTTCAAGGAAGAAAC	geneticin$_{128\text{-}795}$-T$_{trpC}$
Gene_R2 BsaI	TATGGTCTCACCGACCAGTCCTGTTCGTTA	
FgLB_F1	ATATGGTCTCTAACAGTCTCCTTTAATCATGGAGCTGC	LB from PH-1
FgLB AgeI_R	ATATACCGGTTAGTATGGCCCAGCCCTC	
FgLB_F XhoI (P1)[2]	ATATCTCGAGGTCTCCTTTAATC	LB-T$_{trpC}$-geneticin$_{795\text{-}128}$
Gene_R XhoI (P2)[2]	ATATCTCGAGCCGACCAGTCC	
ProTri5_F1	ATATGGTCTCAACCTTCCTAGAACTAAGACATGG	*Tri5* promoter fragment 1
ProTri5_R2	ATATGGTCTCATGTTGATGGCAAGGTTGTACTGG	
ProTri5_R1	ATATGGTCTCAGTCCTTGACGTATGGACGTGCTC	*Tri5* promoter fragment 2
ProTri5_F2	ATATGGTCTCAGGACTCTCTTCACGACTGTCTGG	
ProFgEffector1_F	ATATGGTCTCAACCTTCGGCTATCATACCATCACG	*FgEffector1* promoter
ProFgEffector1_R	ATATGGTCTCATGTTGATGAACGTTTGAAAAAGGTAGTC	
PtrpC_F	ATATGGTCTCAACCTAGTCGCTGCAGGAATTCG	*trpC* promoter
PtprC_R	ATATGGTCTCATGTTTTGGATGCTTGGGTAGAA	
FgEffector1_F	ATATGGTCTCAGGCTGTATGTGCACCCAGGGACTCAAGTA	*FgEffector1* gene
FgEffector1_R	ATATGGTCTCACTGATTTGCCAATGCCTGTTG	
P5	TGCTACAGACAAAACCCGCT	LB genotyping
P6 and P9[1]	GCGACCTACGAGACTGAGGAATCCGCTCTTGG	
P10	AGATGGACTCCCGGACATCA	Combine with P6 for RB genotyping
P11	GTTCATCAAACCCGTTGCCC	To test insertion in TSI locus 1
P12	TTTGAGCCCGATGCAGACAT	
O1	CATGGCGGCCGCGGGAATTCGATTAGACCATTACGGGCCTG	*osp24* promoter
O2	GTTATCGAATAGACTGTGGTGTTTGGATTC	
O3	ACCACAGTCTATTCGATAACTGATATTGAAGGAGCATTTTTT	P$_{trpC}$-HyG$_{1\text{-}761}$
O4	GCCGCGAATTCACTAGTGATGGATGCCTCCGCTCGAAG	
O5	CATGGCGGCCGCGGGAATTCGATCGTTGCAAGACCTGCCTG	Hyg$_{296\text{-}1027}$

O6	CTTGACTCCTCTCGAGGTCGACGGTATC	
O7	CGACCTCGAGAGGAGTCAAGACTGGAATC	*osp24* terminator
O8	GCCGCGAATTCACTAGTGATCACAATAGGAAAGTAAATTGAGATAG	
O9	TTGATCGGTTTCCTGGGAGC	To test *osp24* presence
O10	CACAGTTTCCTCGCGTGTTG	
O11	CATGATATATCACATTCTCGGCGG	*LB genotyping of* PH-1-Δosp24
O12	ACTTCTCGACAGACGTCGC	
O13	ACTCACCGCGACGTCTGT	*RB genotyping of* PH-1-Δosp24
O14	CCAGTTAGTAGCCTGCCCAC	
O15	TGGGTCTCGACCTTCTTACTCTGCAGAAATCAA	*osp24* gene fragment 1
O16	TGGGTCTCGGTCCTAGTTCTCTCTCAATTTAGTC	
O17	TGGGTCTCGGGACTCGCAGGACTGGGATATA	*osp24* gene fragment 2
O18	TGGGTCTCGTAGTCACAATAGGAAAGTAAATTGAGAT	
FGRAMPH1_01T06815F	TGGTCGCATGATGTAACGGG	qPCR amplicon size: 243 bp
FGRAMPH1_01T06815R	GTTGCCATGTTGGTTGTCTCA	
FGRAMPH1_01T06817F	CCACATGAGGCTGGTCACTT	qPCR amplicon size: 162 bp
FGRAMPH1_01T06817R	CAGGTCTGAAGCTGACTGGG	
FGRAMPH1_01T14929F	TCAAGCGTATCTCTGCCATGAT	Histone. qPCR amplicon size: 231 bp
FGRAMPH1_01T14929R	AAGCAGTCGACCGCAAAGA	
FGRAMPH1_01T24551F	CTCCCGAGCGAAAGTACTCC	Actin. qPCR amplicon size: 150 bp
FGRAMPH1_01T24551R	TTGTCGGTCGGGTAGCTTAG	
GFP_F	GGGCACAAGCTGGAGTACAA	qPCR amplicon size: 194 bp
GFP_R	CTCAGGTAGTGGTTGTCGGG	

[1]P9 is the same primer as P6, because a primer that anneals to the *trpC* terminator was select.

[2]Primers in brackets were also used for validation of the transformants.
