# Supplementary figures and images for: Identification and functional characterisation of a locus for target site integration in Fusarium graminearum
Source: Fungal Biol Biotechnol. 2024 Feb 26;11:2. doi: 10.1186/s40694-024-00171-8 (PMC10898126; doi:10.1186/s40694-024-00171-8)

**A**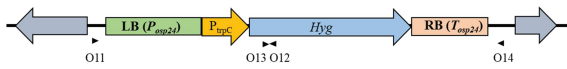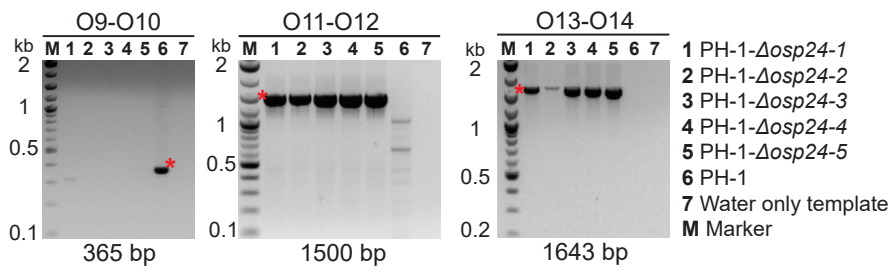**B**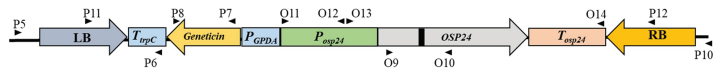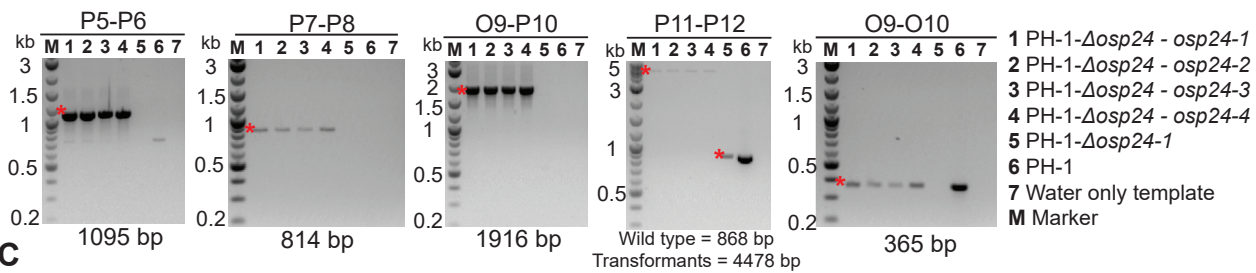**C**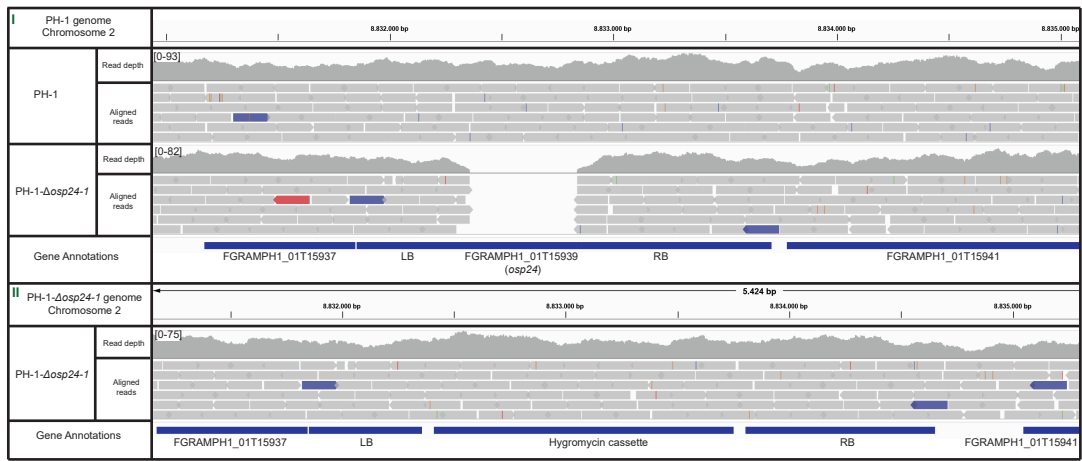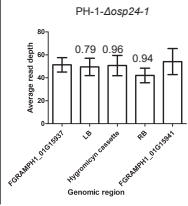**D**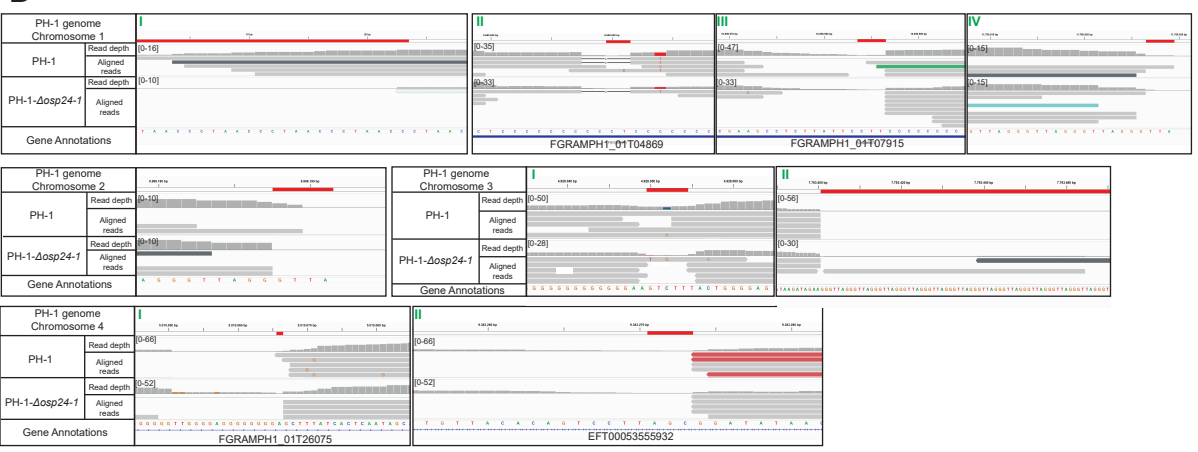

Supplement: Supplementary file 4 — Additional file 4: Genotyping and genomic analysis of PH-1, PH-1-Δosp24 mutant and the Δosp24 complemented strains. A) To select strains where the osp24 gene was deleted, three different PCR products were amplified. Primer combinations O11–O12 and O13–O14 were used to verify the insertion of the Hyg cassette into the osp24 locus. Primer combination O9–O10 evaluates whether the osp24 coding sequence was deleted. B) To select PH-1-Δosp24 complemented strains, five different PCR products were amplified. Primer combinations P5–P6 and O9 and P10 were used to test for correct insertion of the cassette into the TSI locus 1. Primer combinations P7–P8 and P11–P12 were used to evaluated successful recombination between the two PCR fragments and whether each transformant was homokaryotic for the transgene, respectively. Primer combination O9–O10 evaluates the presence of osp24 in the transformed strains. Red asterisks indicate the expected PCR size bands. C) Upper IGV screenshot shows sequencing reads aligned to the FGRAMPH1_01G15939 (osp24) genomic region in PH-1 and PH-1-Δosp24-1. Lower IGV screenshot shows that the coding sequence of osp24 was replaced by the hygromycin cassette. Bar graph (right) represents the average read depth values for the hygromycin cassette and the two genes (FGRAMPH1_01G15937 and FGRAMPH1_01G15941) flanking the osp24 locus. Values above the bars are the ratio value calculated as indicated above. Error bars represent SD of each average coverage value. D) IGV screenshots displaying genomic regions with low read coverage in the PH-1-Δosp24 mutant strain and PH-1. Regions with low coverage were usually identified at the telomeric regions (Chr1I, Chr1IV, Chr2, Chr3II). Other regions such as 5’UTRs (Chr1II, Chr1III and Chr4I) or 3’UTRs (Chr4II) of different genes and an intergenic region (Chr3I) were identified. The red bar above the figure indicates the chromosomic region with read coverage values ≤ 1. Values shown in brackets in the coverage section ar [file 40694_2024_171_MOESM4_ESM.pdf]

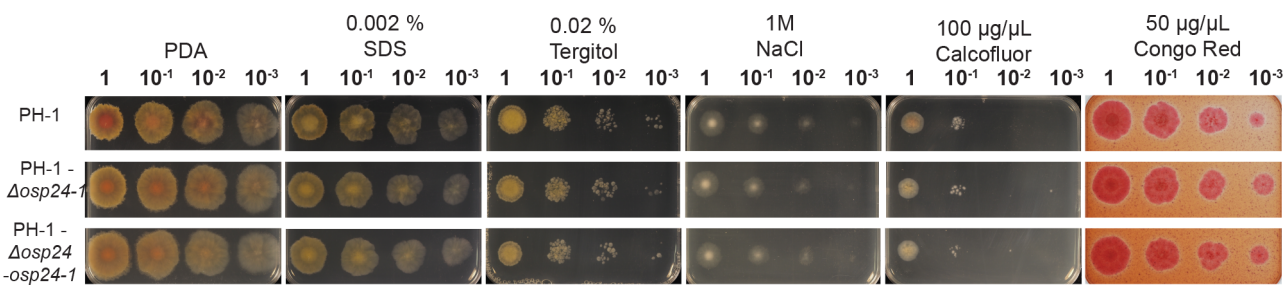

Supplement: Supplementary file 5 — Additional file 5: Stress tests for PH-1, Δosp24 mutant strain and osp24 complemented strain. The mutant strain as well as the complemented strain showed a similar morphology and growth rate as PH-1 for all the conditions tested. Photographs were taken after 3 dpi. Salt stress (NaCl), membrane stresses (Calcofluor, Congo Red, Tergitol, SDS). PDA: potato dextrose agar only. [file 40694_2024_171_MOESM5_ESM.pdf]
