# Supplementary material for: Identification and functional characterisation of a locus for target site integration in Fusarium graminearum
Source: Fungal Biol Biotechnol. 2024 Feb 26;11:2. doi: 10.1186/s40694-024-00171-8 (PMC10898126; doi:10.1186/s40694-024-00171-8)
Supplement: Supplementary file 1 — Additional file 1: Cloning steps to build Fg vector and pJET-LB-Geneticin vectors, and LB and RB sequence alignments. A) A geneticin resistance cassette was built using the Golden Gate cloning approach followed by PCR to amplify a product containing the gpdA promoter and a fragment of the geneticin gene (PgpdA-geneticin1-664). Next, the RB of the TSI locus 1 was amplified from PH-1 genomic DNA. The PCR products from the RB and PgpdA-geneticin1-664 were digested and ligated. During the ligation process a Golden Gate cloning site (CS) was created. A fragment containing a spectinomycin resistance cassette (specR) and a bacterial origin of replication (ori) was amplified by PCR from the pGreen vector. Finally, the specR-ori and the geneticin1-664-PgpdA-CS-RB PCR products were digested and ligated to build the Fg vector. B) From the geneticin cassette a PCR product was amplified containing a fragment of the geneticin gene fused to the TtrpC terminator (TtrpC-geneticin128-795). The LB of the TSI locus 1 was amplified by PCR from PH-1 genomic DNA. The PCR products containing the LB and the TtrpC-geneticin128-795 were digested and ligated. The ligation product was amplified by PCR and cloned into pJET. C) DNA sequence alignments of the LB and RB sequences from PH-1, various other Fg isolates and other Fusarium species. Asterisks indicate positions which have a conserved base among all the isolates and species. F. graminearum (F. gram) isolates: PH-1 (US, GenBank-accession: PRJNA13839), GZ3639 (US, GenBank-accession: PRJNA19849); MDC_Fg1 (France, GenBank-accession: UIHA00000000.2); CS3005 (Australia, GenBank-accession: PRJNA235346); CML3066 (Brazil, GenBank-accession: PRJEB12819) and Fg-12 (China, GenBank-accession: PRJNA743144). F. culmorum (F. culm) UK99 (UK, GenBank-accession: PRJEB12835), F. pseudograminearum (F. pseu) Fp22-2F (China, GenBank-accession: PRJNA871792). F. meridionale (F. meri) JX18-4 (China, GenBank-accession: PRJNA977470) and F. asiaticum (F. asia) KCTC_1 [file 40694_2024_171_MOESM1_ESM.pdf]

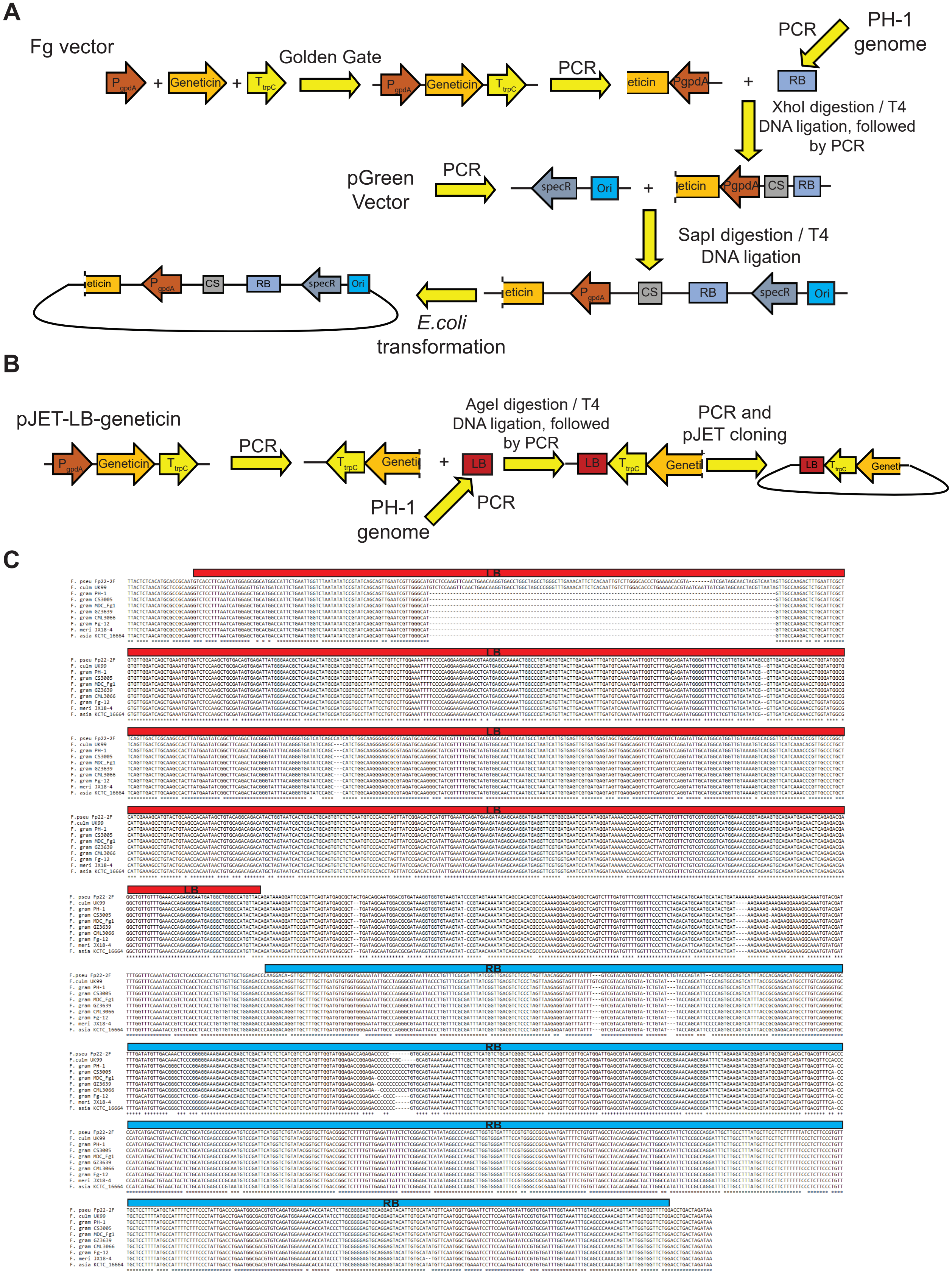
A
Fg vector
P gpdA
Geneticin
T trpC
Golden Gate
PCR
eticin
PgpdA
PH-1 genome
RB
XhoI digestion / T4 DNA ligation, followed by PCR
pGreen Vector
specR
Ori
CS
SapI digestion / T4 DNA ligation
E.coli transformation
B
pJET-LB-geneticin
Geneti
LB
AgeI digestion / T4 DNA ligation, followed by PCR
PCR and pJET cloning
PH-1 genome
C
LB
RB
