# Supplementary material for: Identification and functional characterisation of a locus for target site integration in Fusarium graminearum
Source: Fungal Biol Biotechnol. 2024 Feb 26;11:2. doi: 10.1186/s40694-024-00171-8 (PMC10898126; doi:10.1186/s40694-024-00171-8)
Supplement: Supplementary file 3 — Additional file 3: Genomic sequence analysis of the mCherry expressing strains and PCR amplification of the insertion. A) IGV screenshots displaying genomic regions with low read depth coverage. Regions with low read depth were identified not only in the transformant strains but also in PH-1. Low coverage regions were usually identified at the telomeres (Chr1I, Chr1IV, Chr2, Chr3I, Chr3II and Chr4IV), in the 5’UTRs (Chr1II, Chr1III and Chr4I) or 3’UTRs (Chr4II and Chr4III) of different genes. The red bar above the figure indicates the chromosomic region with read depth values ≤ 1. B) Contigs with evidence of cassette truncations and/or tandem insertions for the mCherry and SP-mCherry strains. Graphs above the contigs represent the read depth for each base of the contig. C) PCR amplification of the insertion at the TSI locus 1 in the mCherry strain. Graph represents the predicted tandem insertion for the mCherry line. [file 40694_2024_171_MOESM3_ESM.pdf]

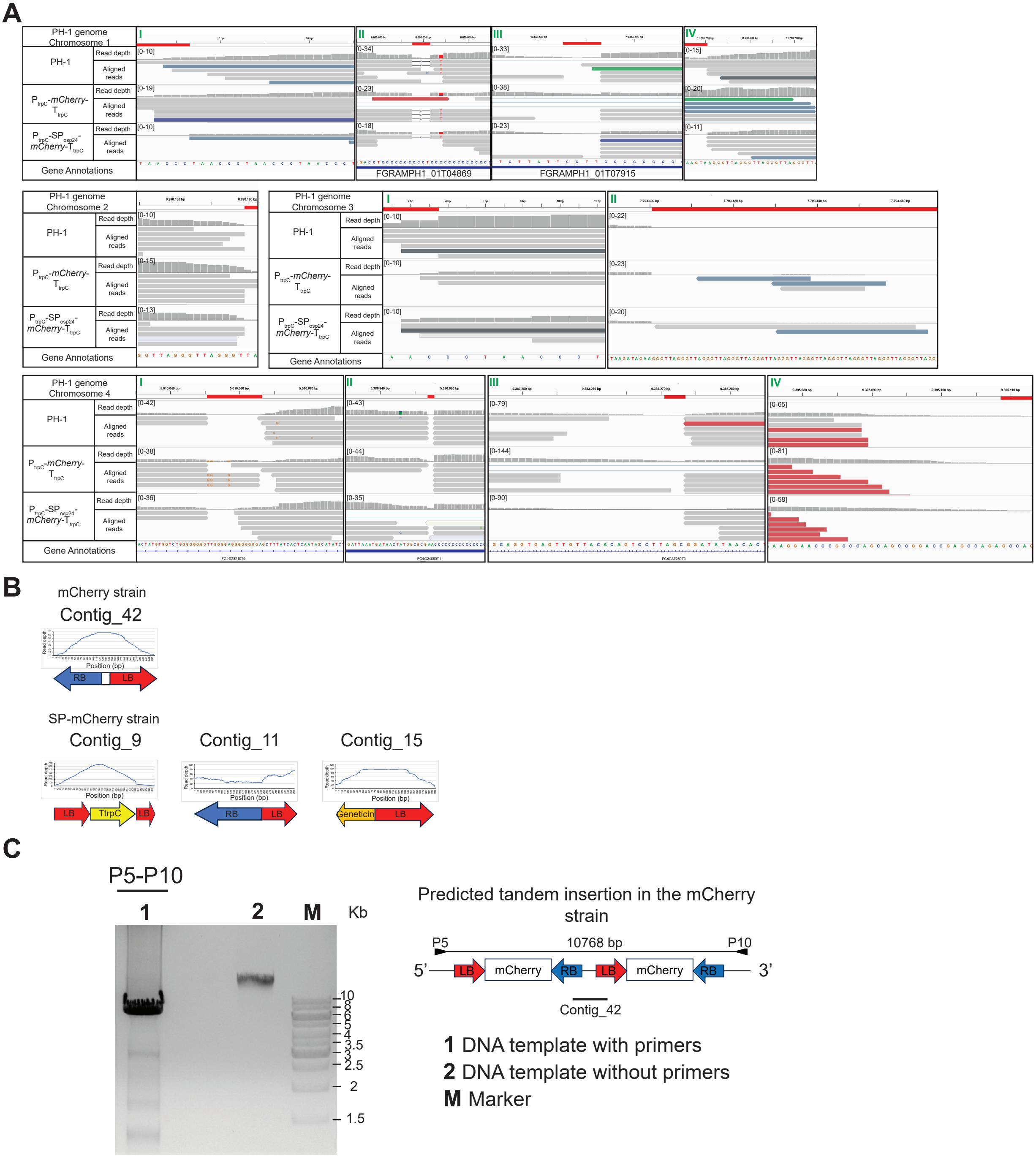
A
PH-1 genome Chromosome 1
PH-1
Read depth
Aligned reads
PtrpC-mCherry-TtrpC
PtrpC-SPosp24-mCherry-TtrpC
Gene Annotations
FGRAMPH1_01T04869
FGRAMPH1_01T07915
PH-1 genome Chromosome 2
PH-1 genome Chromosome 3
PH-1 genome Chromosome 4
B
mCherry strain
Contig_42
Read depth
Position (bp)
RB
LB
SP-mCherry strain
Contig_9
Contig_11
Contig_15
TtrpC
Geneticin
C
P5-P10
1
2
M
Kb
10
8
6
5
4
3.5
3
2.5
2
1.5
Predicted tandem insertion in the mCherry strain
P5
10768 bp
P10
5'
3'
mCherry
Contig_42
1 DNA template with primers
2 DNA template without primers
M Marker
